# Integrated Multifunctional Electronic Skins with Low‐Coupling for Complicated and Accurate Human–Robot Collaboration

**DOI:** 10.1002/advs.202301341

**Published:** 2023-05-17

**Authors:** Chuanyang Ge, Xuyang An, Xinxin He, Zhan Duan, Jiatai Chen, PingAn Hu, Jie Zhao, Zhenlong Wang, Jia Zhang

**Affiliations:** ^1^ State Key Laboratory of Robotics and System Harbin Institute of Technology Harbin 150080 China; ^2^ Key Laboratory of Microsystems and Microstructure Manufacturing Ministry of Education Harbin Institute of Technology Harbin 150080 China

**Keywords:** complicated and accurate human–robot collaboration, low degree of coupling, machine learning, multifunctional electronic skins

## Abstract

Multifunctional capability and low coupling electronic skin (e‐skin) is of great significance in advanced robot systems interacting with the human body or the external environment directly. Herein, a multifunctional e‐skin system via vertical integrated different sensing materials and structures is presented. The multifunctional e‐skin has capacity sensing the proximity, pressure, temperature, and relative humidity simultaneously, with scope of 100–0 mm, 0–30 N, 20–120 °C and 20–70%, respectively. The sensitivity of the four kinds of sensors can be achieved to 0.72 mm^−1^, 16.34 N^−1^, 0.0032 °C^−1^, and 15.2 pF/%RH, respectively. The cross‐coupling errors are less than 1.96%, 1.08%, 2.65%, and 1.64%, respectively, after temperature compensation. To be state‐of‐the‐art, a commercial robot is accurately controlled via the multifunctional e‐skin system in the complicated environment. The following and safety controlling exhibit both accuracy and high dynamic features. To improve the sensing performance to the insulating objects, machine learning is employed to classify the conductivity during the object approaching, leading to set the threshold in dynamic. The accuracy for isolating the insulator increases from 18% to 88%. Looking forward, the multifunctional e‐skin system has broader applications in human–machine collaboration and industrial safety production technology.

## Graphical abstract

E‐skin is significant in advanced robot systems interacting with the human body or the external environment directly. Multifunctional e‐skin with proximity, pressure, temperature, and relative humidity perception at low degree of coupling is proposed here. The sensor array is performed to control commercial robots in human–robot collaboration, which exhibits both accuracy and high dynamic features in following and safety controlling.

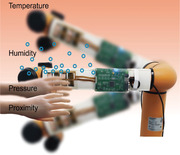

## 1 Introduction

In the last decade, robots have been attempted to employ in collaboration with human in manufacturing,^[^ ^1^, ^2^, ^3^, ^4^ ^]^ medical healthcare,^[^ ^5^, ^6^ ^]^ and housekeeping services.^[^ ^7^, ^8^ ^]^ It is of great significant for robots to collect environmental multiple information to realize accurate, stable, safe, and flexible interaction with people and other objects in complicated environments. However, the current electronic lacks in its multi‐functionality.^[^ ^9^, ^10^, ^11^ ^]^ Human skin is considered to be a complex network that can convert environmental information into electrical signals through various receptors under the skin (baroreceptors, thermal receptors, and pain receptors), and transmit these signals by neural pathways.^[^ ^12^, ^13^, ^14^ ^]^ The neuro‐sexual activity enables human to realize the perception of changes in the complicated environments and makes accurate responses. The mechanical sensory network (e.g., electronic skin, e‐skin) composed of flexible and stretchable sensors can perform as human skin to detect the physiological signals, environmental stimuli, and body motions effectively.^[^ ^15^, ^16^ ^]^ Inspired by this, the e‐skin has also been developed for perception of the environmental information for robot control.^[^ ^13^, ^17^, ^18^, ^19^, ^20^ ^]^

The multi‐functionalities, high accuracy, and low coupling are of essential importance in developing e‐skin to mimic the function of human skin.^[^ ^13^, ^21^ ^]^ However, previous researches are mainly focused on the single‐ or dual‐functions.^[^ ^22^, ^23^, ^24^, ^25^ ^]^ Many sensors have exhibited excellent performance based on the piezoelectric,^[^ ^26^, ^27^ ^]^ thermal,^[^ ^28^, ^29^, ^30^ ^]^ triboelectric,^[^ ^31^, ^32^, ^33^ ^]^ capacitive,^[^ ^25^, ^34^ ^]^ electromagnetic,^[^ ^35^, ^36^ ^]^ and optical mechanisms.^[^ ^37^, ^38^ ^]^ Recently, many attempts have been made to realize multi‐information perception. For instance, a deformable artificial multimodal ionic receptor can differentiate thermal and mechanical information without signal interference.^[^ ^24^ ^]^ The multimodal ionelectronic skin, which consists of the receptor, provides real‐time force directions and strain profiles in various tactile motions (shear, pinch, spread, torsion, and so on). A resultant functional (PVDF‐TrFE) fiber mat gives rise to an anisotropic in‐plane conductive network for in‐plane strain sensing, while the oriented ferroelectric crystals in nanofibers with piezoelectricity allow for out‐of‐plane dynamic pressure detection.^[^ ^39^ ^]^ A self‐protected piezoelectric–piezoresistive dual‐mode device achieves static/dynamic pressure sensing with high sensitivity by vertically arranging the two modes, which shows the ability to be suitable for more complex application scenarios.^[^ ^40^ ^]^ Above all, multi‐information perception can be divided into three categories. The first one is to develop functional materials and structures, which can be sensitive to various environmental information.^[^ ^41^, ^42^, ^43^ ^]^ The second one is to decouple perception information from a single measurement via the signal post processing.^[^ ^44^, ^45^ ^]^ However, both methods have a strong coupling effect between different information. It is highly necessary to design the effective decoupling algorithms to extract specific environmental information. Up to now, many attempts have been done in decoupling process, however, the calculations are still complex and time‐consuming. What is more, the decoupling accuracy and result are still far from satisfactory. It is in this setting that the third one is to directly integrate different sensing materials and structures in plane or stacking features.^[^ ^46^, ^47^, ^48^ ^]^ In this scenario, the coupling of multi‐information taken from e‐skin could be reduced to its limitation. The inevitable coupling information (e.g., temperature and pressure) can be isolated effectively first, and then compensated accordingly.^[^ ^34^, ^49^ ^]^ Nevertheless, this attempt still has complicated structures and complex process flow as well as signal conditioning, which will be a time‐consuming and costly method.^[^ ^47^ ^]^

Here, we present an integrated multifunctional e‐skin that is capable of detecting proximity, pressure, temperature, and humidity simultaneously. The multifunctional sensing capacities are revealed with the proximity distance, contact pressure, object temperature, and environmental relative humidity range of 100–0 mm, 0–30 N, 20–120 °C, and 20–70%, respectively. The sensitivity of above four kinds of sensors can be achieved 0.72 mm^−1^, 16.34 N^−1^, 0.0032 °C^−1^, and 15.2 pF/%RH, respectively. The cross‐coupling errors of the four kinds of information after temperature compensation are below 1.96%, 1.08%, 2.65%, and 1.64%, respectively. To be state‐of‐the‐art, a commercial robot has been controlled with the multifunctional e‐skin system in a complicated environment, which exhibits to not only realize interrupt control for different environmental changes but also perform real‐time dynamic follow‐up response to approach and pressure accurately. Finally, the machine learning is employed to optimize the performance of the target approach process, thereby improving the ability, especially for the insulating object, to deal with following control and active safety control in complex environments.

## 2 Results and Discussion

### 2.1 Design of Integrated Multifunctional E‐Skin

**Figure 1:**
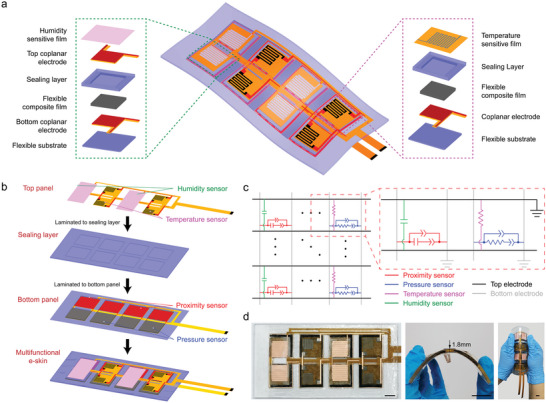
Multifunctional electronic skin (e‐skin) with capacity of sensing proximity distance, contact pressure, temperature, and relative humidity. a) Schematic illustration of the multifunctional e‐skin, where the left and right panel are two types of sensing units. b) Integrated fabrication process for the multifunctional e‐skin. c) Circuit diagram of the sensor array. d) The pictures of the multifunctional e‐skin, the scale bars are all 10 mm.

To realize accurate operation of robot and precise human–robot collaboration (HRC), a multifunctional e‐skin is highly required to acquire environmental information. **Figure** **1**a schematically illustrates the designed e‐skin for multi‐information acquirements. In brief, the left panel exhibits an enlarged explosive view of the humidity/proximity/pressure sensor unit, which contains a humidity sensitive film, a top coplanar electrode, a silicone sealing layer, a flexible composite film formed by carbon black fillers in the polydimethylsiloxane (PDMS) matrix, a bottom coplanar electrode, and a silicone flexible substrate. The right panel exhibits an enlarged explosive view of the temperature/proximity/pressure sensor unit, which contains five layers with a serpentine nickel film as the temperature sensitive layer instead of the top coplanar electrode and humidity sensitive film (Figure 1a left panel). Figure 1b illustrates the fabrication process of the multifunctional e‐skin. The top coplanar electrode contains four comb‐shape electrode units and four blank units. A serpentine nickel film with a thickness of 100 nm was sputtered on the four blank units, which forms the temperature sensor (Figure S1a, Supporting Information). The humidity sensitive film made by Polyvinylidene fluoride/ Polyvinyl alcohol/Lithiumchloridemonohydrate (PVDF/PVA/LiCl) (Figure S1b, Supporting Information) is attached to the comb‐shape electrode units in the top electrode by silicone rubber. The bottom coplanar electrode (Figure S1c, Supporting Information) is pasted on the flexible substrate (Figure S1e, Supporting Information), which contains eight comb‐shape electrode units. Flexible composite stripes (Figure S1d, Supporting Information) are placed over comb‐shape electrodes to form proximity sensors and pressure sensors. Prior to lamination, the interfaces of the sealing layer (Figure S1f, Supporting Information), flexible composite films and flexible substrate are implemented to oxygen plasma treated to promote adhesion properties. Finally, a multifunctional e‐skin is fabricated successfully.

Figure 1c illustrates an equivalent circuit diagram of the multifunctional sensing array. The adjacent two units consist of four different sensors: one is a capacitive humidity sensor based on humidity‐sensitive material on the top layer (green), and the corresponding bottom layer is a capacitive proximity sensor based on a flexible composite film (red); the other is a resistive temperature sensor based on nickel film on the top layer (pink), and the corresponding resistive pressure sensor based on flexible composite film (blue). The top sensor is between the black lines, while the bottom sensor is between the gray lines (Figure 1c). The resistive sensors are measured using a Direct Current (DC) input, whereas the impedance and capacitive sensors are tested using an Alternating Current (AC) input. After determining the bonding method and equivalent circuit diagram, the multifunctional flexible e‐skin is assembly fabricated as shown in Figure 1d. The final dimension of e‐skin is 144 × 74 × 1.5 mm^3^, which will be used in the HRC further. Large‐angle deformation of e‐skin shows its good bendability and adhesion (Figure 1d right panel).

### 2.2 Sensing Performances of the Temperature/Proximity/Pressure Sensor Unit

**Figure 2:**
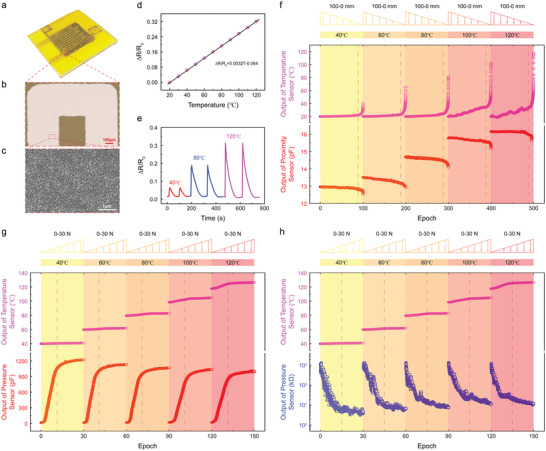
Sensing performances of the temperature/proximity/pressure sensor unit. a) The picture of the temperature/proximity/pressure sensor unit. b) Optical image of the spiral nickel film for temperature sensing. c) SEM image of the nickel film surface. d) The plots of Δ*R*/*R*_0_ with respect to the temperature. e) The continuous response (Δ*R*/*R*_0_) of the temperature sensor at 40, 80, and 120 °C, respectively. f) Simultaneous and independent detections of object temperature (top) and proximity distance (bottom). g) Simultaneous and independent detections of object temperature (top) and contact pressure in capacitive mode (bottom). h) Simultaneous and independent detections of object temperature (top) and contact pressure in resistive mode (bottom).

**Figure** **2**a shows a temperature sensor unit with a thickness of 100 nm serpentine nickel film as the sensitive material and structure (Figure 2b). The intact surface demonstrates a good adhesion of the nickel film to the substrate (Figure 2b). Scanning electron microscope (SEM) image of the nickel film after annealing (Figure 2c) shows a uniform surface morphology with increased grain size compared with its original state once after sputtering deposition (Figure S2a, Supporting Information). The above changes lead to a significant improvement sensing stability of temperature (Figure S2b, Supporting Information).^[^ ^50^ ^]^ The final dimension of the sensor unit is 30 × 30 × 1.5 mm^3^, which is one kind of unit of 4 × 2 array in e‐skin. A thin polyimide (PI) film is used to encapsulate the above temperature sensitive film to avoid abrasion or oxidation in air.

Figure S3a, Supporting Information, plots a linear increasing of resistance as the temperature increases from 20 to 120 °C without pressure, from which the resistance variations (Δ*R*/*R*_0_) are calculated and presented in Figure 2d. The linear fitting curve results in a relationship of Δ*R*/*R*_0_ = 0.0032*T* − 0.064 (Figure 2d). The straight slope is 0.0032, which is equal to the temperature sensitivity in number. As‐prepared nickel temperature sensor exhibits an excellent linearity, high sensitivity, and large response range compared with other temperature‐sensitive materials.^[^ ^51^, ^52^, ^53^ ^]^ Figure 2e sequentially shows the continuous response and recovery of the temperature sensor at 40, 80, and 120 °C, respectively. The response time is about 1.5 s at each temperature, while the recovery times are 75, 105, and 125 s accordingly. In addition, we conducted continuous response and recovery experiments at 20 and 0 °C. The relative change of the temperature sensor is about 4%. The response time is about 1.5 s, while the recovery time is about 90 s (Figure S3b, Supporting Information). Figure S3c, Supporting Information, plots the resistance change with the temperature fluctuating at 30 and 120 °C repeatedly for 54 000 s, which exhibits a very stable sensor output. In addition, the resistance change at the last 480 s is compared with its initial 480 s, resulting in the same level (Figure S3b, Supporting Information, inset). The stability of resistance plays a significant role in the actual usage on the repeated temperature perception.

Since the temperature sensor is directly attached upside of the temperature/proximity/pressure sensor unit, there is highly necessary determination of detection three information in simultaneous and independent. Figure 2f plots the results of the temperature and proximity sensor outputs simultaneously. It is noteworthy that the temperature sensor output keeps the same as the proximity distance of the object beyond 10 mm, while it increases gradually as the proximity distance within 10 mm (Figure 2f). The tendency is more significant as the temperature above 80 °C since the heat transferring in air between the large temperature difference objects is more efficient and obvious. The temperature change rate reaches the maximum value as the object contacting to the sensor (Figure S4a, Supporting Information). Meanwhile, the proximity sensor output increases with temperature increasing since the polydimethylsiloxane (PDMS) does not have good thermal insulation properties. Figure S4b, Supporting Information, plots the original value of the proximity sensor with respect to the object temperature. The increase in temperature leads to a decrease in the spacing between the conductive fillers inside the flexible composite film, resulting in enhanced dielectric properties. An increase in the relative permittivity causes the original value of the proximity sensor to increase as the temperature of the object increases with a cubic polynomial formula as Equation (1).

(1) $$C=16.78-0.23T+0.0035T^{2}-1.51\times 10^{-5}T^{3}$$

The relative change rate of capacitance during the object approaching also shows the similar trend as above. The conductive fillers in the flexible composites are thermally perturbed by heating, resulting in changes in the electric field between the carbon blacks. The kinetic energy obtained by the conductive filler increases, which increases the probability of collision and connection among the carbon black particles. The proximity sensor output increases as the dielectric constant of the flexible composite material increases. The above formula can be used as the temperature compensation coefficient in the proximity process during designing of the capacitor–resistance data acquisition circuit.

Figure 2g,h plots the coupling effect of the temperature/proximity/pressure sensor unit in simultaneously detecting environment temperature, object proximity, and contact pressure. The temperature sensor output increases as the pressure increases (Figure S5a, Supporting Information). In this scenario, the nickel film is stretched and deformed under the applied pressure, which may increase the sensor resistance. However, the resistance change rate within 30 N at different temperatures is less than 2.56% (Figure S5b, Supporting Information), indicating a low‐coupling between temperature and pressure (<30 N). Furthermore, the capacitance increases significantly with increasing pressure and saturates after the pressure exceeding 15 N. The initial capacitance value is collected at the proximity distance of 0 at each corresponding temperature. The resistance decreases significantly with the increase of pressure, and the output shows a piecewise linear law under logarithmic coordinates. The maximum sensitivity is calculated to be 16.34 N^−1^, which is of great magnitude for the tactile sensors based on the PDMS composites.^[^ ^54^, ^55^, ^56^, ^57^, ^58^ ^]^ The saturation capacitance value of the pressure sensor decreases with the increase of temperature, while the minimum resistance value increases with the increase of temperature. The saturation capacitance and minimum resistance with respect to temperature are shown in Figure S5c,d, Supporting Information, which can be calculated as

(2) $$C=1594.19-13.19T+0.12T^{2}-3.83\times 10^{-4}T^{3}$$

(3) R=0.10T+0.18

The phenomenon can be explained by the tunnel effect. The environment temperature causes the pressure sensor to be heated, leading to expansion of the polymer matrix in the flexible composite material. The distance between the carbon black particles increases upon the external force, which is induced by a slippage of the conductive particles inside the composite material, leading to destruction of the conductive network. The resistivity increases and the relative permittivity decreases, if the destruction speed is faster than the path formed. Equations (2) and (3) are also used as the temperature compensation coefficient in the contact process in the design of the capacitor–resistance data acquisition circuit. Figure S6, Supporting Information, plots the variation of resistance at 100°C with repeated pressure fluctuations at 0 and 30 N. The drift rate of resistance is 1.64%, indicating a good pressure stability of the temperature sensor. Figure S7a,b, Supporting Information, plots the variation of capacitance and resistance of pressure sensor of temperature/proximity/pressure sensor unit at 120 °C with repeated pressure fluctuations at 0 and 30N. The drift rates are 1.45% and 3.28%, respectively, which shows a good pressure stability of the pressure sensor.

### 2.3 Sensing Performances of the Humidity/Proximity/Pressure Sensor Unit

**Figure 3:**
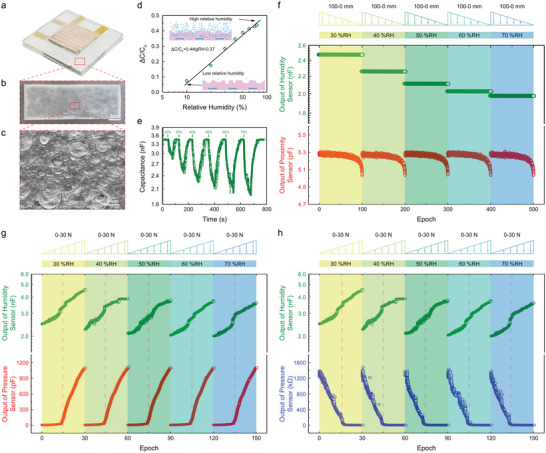
Sensing performances of the humidity/proximity/pressure sensor unit. a) The picture of the humidity/proximity/pressure sensor unit. b) The picture of the humidity sensitive film. c) SEM image of the surface of the humidity sensitive film. d) The plots of |Δ*C*|/*C*_0_ of the humidity sensor with respect to the relative humidity. e) The continuous response (|Δ*C*|/*C*_0_) of the humidity sensor at the relative humidity of 20%, 30%, 40%, 50%, 60%, and 70%, respectively. f) Simultaneous and independent detections of environment relative humidity (top) and proximity distance (bottom). g) Simultaneous and independent detections of environment relative humidity (top) and contact pressure in capacitive mode (bottom). h) Simultaneous and independent detections of environment relative humidity (top) and contact pressure in resistive mode (bottom).

**Figure** **3**a shows a sensor unit with a humidity sensitive film (Figure 1a left panel). The dimension of the sensor unit is 30 × 30 × 1.5 mm^3^, which is another kind of unit of 4 × 2 array in e‐skin. Here, the humidity sensitive film is bare in environment without sealed layer. The humidity sensitive material is attached on the top coplanar electrode with the size of 30 × 30 × 0.1 mm^3^, which is cut from the film shown in Figure 3b. Figure S8, Supporting Information, exhibits the fabrication steps of the humidity sensitive film, in which the film exhibits white and slightly transparent with a thickness of 80 µm. Figure 3c shows the enlarged SEM image of the surface of the humidity sensitive film, which is composed by a large number of micro‐pores with a diameter of 5–50 µm. The micro‐pores can carry out the adsorption water molecules and transport them very well by avoiding the agglomeration of water molecules. This structure can make the sensitive film adsorb water molecules evenly (upper panel in Figure 3d), and desorb as much as possible during dehydration (lower panel in Figure 3d). Therefore, it will reduce the hysteresis in humidity sensing. It can be found that the micro‐pores do not completely penetrate the entire cross‐section from the cross‐sectional view of the humidity sensitive materials (Figure S9, Supporting Information). This structure can avoid baseline drift effectively, which is caused by the electrode coming into contact with moisture.

The water contact angle of the PVDF/PVA/LiCl humidity‐sensitive film is 85.90° (Figure S10, Supporting Information), indicating that the material has a good hydrophilic property and is suitable for the fabrication of humidity sensitive film. Figure 3d plots the capacitance change rate with respect to relative humidity (RH), resulting in linear relationship in the logarithmic coordinate. The capacitance decreases from 3.25 to 2.05 nF as the RH increases from 10% to 80% (Figure S11a, Supporting Information). The large base value can minimize the influence of ambient noise and parasitic capacitance on humidity perception effectively. Herein, the absolute value of the capacitance change is used for calculating the capacitance variations (Δ*C*/*C*_0_). The absolute value of the capacitance change gradually increases. Therefore, the capacitance variations (Δ*C*/*C*_0_) increase as the RH increases without pressure. The experimental data can be fitted with a line in logarithmic coordinates (Equation 4), leading to a straight slope of 0.4363 (Figure 3d).

(4) $$\Delta C/C_{0}=0.44\mathrm{lg}\mathrm{RH}-0.37$$

Accordingly, the sensitivity of humidity sensor is equal to 15.2 pF/%RH with a linearity of 95.6%. The capacitance of the humidity sensor can be considered to be composed of the water vapor film and the humidity sensitive material in parallel. The composite dielectric constant will be changed while the water molecules adsorbs into the humidity‐sensitive film. Figure 3e sequentially shows the continuous response of the humidity sensor at 20% and 70%. The response and recovery time is about 40 and 30 s at RH of 20%. In the high RH, the convective transfer rate is faster compared with that of low RH (e.g., 20%) due to large humidity difference to RH of 0. As a result, the recovery time is not proportional to RH. Figure S10b, Supporting Information, plots the capacitance change with the RH fluctuating at 0% and 70% repeatedly, which exhibits a very stable output. We also compare the capacitance change at the last 1500 s with its initial 1500 s, resulting in the same level (Figure S11b, Supporting Information, inset). The stability of capacitance plays a significant role in the usage on the repeated humidity perception.

For detection of the humidity, proximity distance, and contact pressure simultaneously, the coupling properties is highly important. Figure 3f shows the results of the humidity/proximity/pressure sensor unit in detecting environment humidity and proximity distance simultaneously. For the humidity sensor, the output difference with the distance of 100–0 mm is only 0.5% (Figure S12a, Supporting Information), so that the cross‐coupling error of humidity sensing for proximity distances is less than 0.5%. For the proximity sensor, the initial outputs at different RH are all about 5.27 pF, while the outputs are all about 5.0 pF in the critical state (Figure S12b, Supporting Information). The electric field line between the bottom electrode and the object is partially attracted by the top electrode because of the inevitable shielding effect to bottom coplanar electrode by the top coplanar electrode, resulting in the decrease of the mutual capacitance of the bottom electrode. Both the basic value and the variation of the tactile sensor are reduced compared with previous reported results.^[^ ^59^ ^]^ It is because of the hydrophobicity of PDMS that moisture cannot enter the interior of the sensor unit or adhere to other surfaces except the humidity sensitive film. The relative dielectric constant of each component inside the sealing layer does not change. The output of the proximity sensor does not change with humidity. The cross‐coupling error of proximity sensing for RH is considered to be less than 2%.

Figure 3g,h plots the coupling effect of the humidity/proximity/pressure sensor unit in simultaneously detecting environment RH and contact pressure. For the humidity sensor, the micropores on the surface of the humidity sensitive layer are subjected to the tension and deformation upon the pressure. The water molecules originally adsorbed on the surface and side walls of the micropores deabsorb. Meanwhile, the contact area between the water molecules and the micropores decreases. As a result, the actual contact surface of water molecules decreases and the capacitance value of the humidity sensitive layer increases^[^ ^60^, ^61^ ^]^ (Figure S13a, Supporting Information). The relationship between the humidity sensor output and the contact pressure can be fitted as *C* = 72.60*F* + 2309.45.

The linear relationship can be explained by the desorption of water molecules pre‐adsorbed on the surface and sidewalls of the micro‐pores upon the stretched and deformed under the force. Simultaneously, the contact area between the water molecules and the micro‐pores decreases, leading to a decrease of RH on the surface, thereby the humidity sensor output increases. The relative capacitance change rate of the humidity sensor capacitance is a little fluctuation around 82.5% at 30N under the RH in the range of 30–70% (Figure S13b, Supporting Information). The capacitance change caused by the humidity and pressure has a totally opposite tendency, which makes it easy to distinguish one from the other. For the pressure sensor, the capacitance increases significantly with increasing pressure, whereas the resistance shows an opposite tendency. Both modes exhibit a piecewise linearity at both sides of the cut‐off point (15 N), in which the sensitivity of capacitive mode and resistive mode are 0.68 and 13.06 N^−1^ in 0–15 N, while they are 16.27 and 0.45 N^−1^ in 15–30 N, respectively. The errors for the above capacitance and resistance modes are only 3.47% and 3.81% compared with the simulation results.^[^ ^62^ ^]^ The moisture in air is isolated by the PDMS sealing layer, so that it does not change the conductive path distribution of the flexible composite. Therefore, the pressure sensor output changes only with pressure. The maximum capacitances are all about 1101.5 pF with RH in the range of 20–70% (Figure S13c, Supporting Information), while the minimum resistances are all about 2.62 kΩ (Figure S13d, Supporting Information). The cross‐coupling error of pressure sensing in capacitive mode under the RH is considered to be less than 0.98%, while it is less than 4.36% in resistive mode. Figure S14, Supporting Information, plots the variation of capacitance at RH of 70% with repeated pressure fluctuations at 0 and 30 N. The capacitance drift rate is 1.08%, indicating a good stability of the humidity sensor under the pressure. Figure S15a,b, Supporting Information, plots the variation of capacitance and resistance of pressure sensor of humidity/proximity/pressure sensor unit at 70% with repeated pressure fluctuations at 0 and 30N. The drift rates are 1.09% and 2.76%, respectively, which shows a good pressure stability of the pressure sensor. Finally, the device's performances are compared to the previous reported results and the results are accumulated in Table S1, Supporting Information. Impressively, as‐prepared devices show significant large distance and force range scopes than others.

### 2.4 The Coupling‐Effect of the Two Types of Units

To realize multi‐information detection, two kinds of sensing units are vertically integrated in array forming an e‐skin (Figure 1d). The e‐skin can be responsive to distance, temperature, humidity, and pressure signals based on the capacitance and resistance modes. To end this, a home‐made capacitance–resistance data acquisition circuit is developed to collect data at the same time. Figure S16a, Supporting Information, illustrates the principle diagram for the sensor measurement, while the test board is displayed in Figure S16b, Supporting Information. The coverage of capacitance and resistance measurement is in 0.01 pF–10 nF and 0.1 kΩ–1 MΩ. The static sensor performances are measured in a home‐made platform (Figure S17, Supporting Information), which is also used to study the mutual crosstalk between the two types of four units of the sensing array under static conditions.

**Figure 4:**
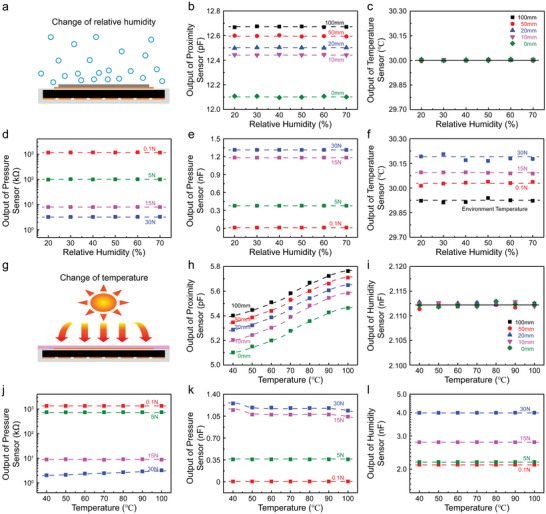
The coupling‐effect of the two types of units. a) Schematic illustration of temperature/proximity/pressure sensor unit influenced by moisture. b) Performance of the proximity sensor influenced by relative humidity. c) Performance of the temperature sensor during the object approaching influenced by relative humidity. d) Performance of the pressure sensor in resistive mode influenced by relative humidity. e) Performance of the pressure sensor in capacitive mode influenced by relative humidity. f) Performance of the temperature sensor in contacting object influenced by relative humidity. g) Schematic illustration of humidity/proximity/pressure sensor unit influenced by object temperature. h) Performance of the proximity sensor influenced by object temperature. i) Performance of the humidity sensor during the object approaching influenced by its temperature. j) Performance of the pressure sensor in resistive mode influenced by the object temperature. k) Performance of the pressure sensor in capacitive mode influenced by the object temperature. l) Performance of the humidity sensor in contacting object influenced by its temperature.

**Figure** **4**a–f shows the performance of temperature/proximity/pressure sensor unit affected by RH. Figure 4a illustrates the test diagram with RH in 20–70%. The capacitance output error of the proximity sensor with respect to RH at each distance (0, 10, 20, and 50 mm) is less than 0.58% (Figure 4b), indicating the performance proximity sensor is not influenced by humidity. The resistance output error of the temperature sensor under the different RH at each distance is less than 0.4% (Figure 4c) at the object temperature of 30 °C, indicating the temperature detection is not affected by the RH and proximity distance. The resistance (Figure 4d) and capacitance (Figure 4e) changes of the pressure sensor under different RH are consistent with the performances of pressure sensor in Figures 2h and 2g, respectively. The cross‐coupling error of the temperature sensor with respect to RH under the pressure is only 1.37%, which proves that the temperature sensor is not affected by humidity (Figure 4f). The low‐coupling effect can be also explained by the PDMS sealing layer, which can isolate the device from the moisture in air. Therefore, the relative dielectric constant of each part does not change, resulting in the same capacitance.

Figure 4g–l shows the performance of humidity/proximity/pressure sensor unit affected by the object temperature. Figure 4g illustrates the measurement diagram, in which the object temperature is set 40–100 °C. The capacitance at the same proximity distance increases with the temperature (Figure 4h) and the change rule is given by Equation (1). The trend at the same temperature is consistent with that in Figure 3f. The capacitance output error of humidity sensor under the different object temperatures at the same distance is less than 2.59% (Figure 4i), which indicates that the humidity detection is not affected by object temperature and proximity distance. The resistance (Figure 4j) and capacitance (Figure 4k) changes of the pressure sensor under different object temperatures are consistent with the performance of pressure sensor in Figures 3h and 3g, respectively. The output value does not change as the temperature in a tiny force (<5N). However, the relationship between the output value and the temperature in considerable pressure is consistent with Equations (2) and (3). Accordingly, the temperature compensation on the resistance of the pressure sensor is performed as designing the test circuit of the sensor array. As a result, the cross‐coupling error of the humidity sensor with respect to object temperature under the pressure is only 0.5%, which proves that the humidity sensor is not affected by the object temperature. The performance variation of the proximity and pressure sensors is caused by the thermal conductivity of PDMS, whereas humidity sensitive films are not affected by temperature. The sensor units of the e‐skin are adjusted and extended to predefined locations for multifunctional perception. The e‐skin is fabricated layer‐by‐layer, in which the structure of each layer and the fabrication process are feasible and simple, so that a low degree of coupling with each other can be achieved. It can be used to emulate the different densities of mechanoreceptors in the human skin through network sensing area expansion and array design.

### 2.5 Motion Control of the Robot Arm Equipped with Multifunctional E‐Skin System

**Figure 5:**
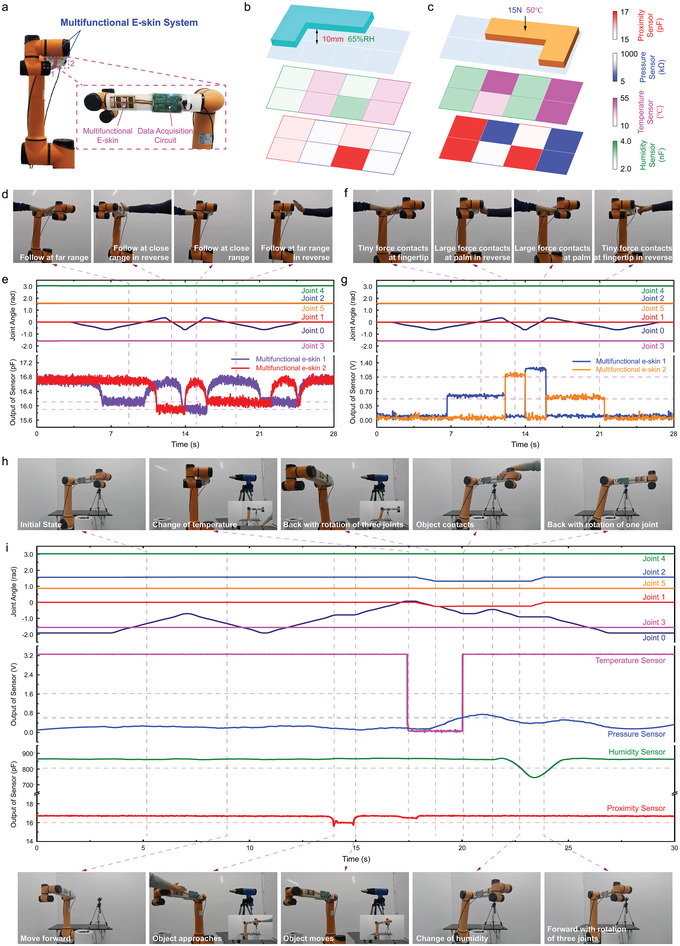
Motion control of the robot arm (AUBO, i‐10) equipped with multifunctional e‐skin system. a) The picture of the multifunctional e‐skin system equipped on robot arm. b) Electrical signal mapping of the e‐skin with a “L‐shape” stainless steel block at distance of 10 mm and the relative humidity of 65%. c) Electrical signal mapping of the e‐skin with an “L‐shape” stainless steel block upon a contact pressure of 15N at the temperature of 50 °C. d) The snapshots of robot arm collaboration with human palm under the distance following control based on the proximity sensor. e) The proximity sensor output and joint angle during the human palm approaching under the distance following control. f) The snapshots of robot arm collaboration with human palm under pressure following control based on the contact force sensor. g) The force sensor output and joint angle upon the human finger and palm contacting under the pressure following control. h) The snapshots of robot arm collaboration with human palm and finger as well as environmental moisture and heat. i) The proximity, pressure, temperature, humidity sensor output, and joint angle based on the proximity distance, contact pressure, object temperature, or environmental humidity change.

For practical applications, the multifunctional e‐skin and data acquirement system are attached to the commercial robot arm (**Figure** **5**a, i10, AUBO). The complicated information in environment has been acquired accurately for the active control robot in HRC. Figure S18, Supporting Information, illustrates the multifunctional e‐skin application platform, which contains the robot arm, a temperature generating device, a humidity generating device, and the e‐skin system. Herein, the e‐skin system contains a multifunctional e‐skin and data acquirement circuit. They are attached to the farthest link of the robot arm in a back‐to‐back arrangement (Figure 5a). According to the cross‐coupling performance between the sensors mentioned above, the sensor input matrix interface is designed (Figure S19, Supporting Information). The design of matrix interface and temperature compensation can effectively reduce the interference between each information. Figure 5b illustrates electrical signal mappings of the sensor array when an L‐shaped stainless steel block approaches at distance of 10 mm with the RH of 65%. The proximity sensor output units corresponding to the L‐shaped block decreases, while the other units keep unchanged. Meanwhile, the pressure sensor units do not change either. Analogously, the humidity sensor units output corresponding to the L‐shaped block decreases, while the other units do not change. In addition, the temperature sensor units do not change. It demonstrates the excellent proximity and humidity sensing capabilities as well as the low‐coupling with respect to temperature and pressure. Figure 5c plots electrical signal mappings of sensor array when an L‐shaped stainless steel block contacts to the e‐skin under the pressure of 15 N at the temperature of 50 °C. The pressure sensor units output corresponding to the L‐shaped block decreases, while the other units keep unchanged. The proximity sensor unit's outputs do not change either. Meanwhile, the output value of the temperature sensor units corresponding to the L‐shaped block increases, while the other units do not change. The humidity sensor unit's outputs do not change. Since the output of the proximity sensor unit and the humidity sensor unit has an opposite changing trend under the pressure, there is no data transmitted to the host computer after being judged by data acquisition circuit. The pressure sensor unit and the temperature sensor unit can obtain accurate values of corresponding information respectively through temperature compensation and pressure compensation.

Figure 5d,f shows snapshots of robot and human arms collaboration via the multifunctional e‐skin system. For the distance following control, the motion of the robot is controlled under different proximity distances (Movie S1, Supporting Information). As shown in Figure S20, Supporting Information, the robot arm moves away at speed of 9° s^−1^ with distance of 40 mm, while moves away at 18° s^−1^ with distance of 20 mm. Figure 5e plots the proximity sensor outputs of e‐skin and the inner joint angle at the same time. Herein, the safety distance for the object proximity is set at 40 mm, which results in the relative change of capacitance below 2.75%. It is considered as the threshold for controlling movement of robot arm. The robot arm moves forward normally as the sensor output above the threshold, while lowers the speed as the output below threshold. The capacitance is below 16.1 pF at the proximity distance above 40 mm, while it decreases to 15.9 pF at 20 mm. For the following control of the robot arm via the pressure, the movement of the robot arm is controlled under different contact pressures (Movie S2, Supporting Information). As shown in Figure S21, Supporting Information, the robot arm moves away at speed of 9° s^−1^ with a tiny force, while moves away at 18° s^−1^ with a considerable pressure. Figure 5g plots the pressure sensor outputs of e‐skin and inner joint angle at the same time. Here, a tiny force is applied with fingertips, while a considerable pressure is applied by palm. When the force is greater than 0.5 N, the output voltage is larger than 0.52 V. Meanwhile, the robot arm moves in reverse at a slow speed (i.e., 9° s^−1^). What is more, the robot arm moves away from the object quickly with the pressure larger than 10 N, in which the output voltage increases up to 1.05 V. The above process verifies the dynamic performance, fast response, and recovery performance of each unit of the multifunctional e‐skin system and clear spatial resolution as well.

For the active safety control for various environmental changes, the motion of the robot arm has been controlled by the posture changes when it encounters the different environments (i.e., object approaching, contact force, humidity change, or temperature change) (Movie S3, Supporting Information). Figure S22, Supporting Information, shows a flowchart of active controlling robot arm via various environmental changes. In brief, the robot arm stands until the object approaching before the set distance (i.e., 20 mm) and moves away within 20 mm. The robot arm moves away from heat source as the temperature is more than 50 °C. Robot arm stands for 1s and then moves away upon the contacting of the object contacts. Furthermore, the robot arm returns to positive position with the RH changes to 60%. Figure 5i plots the e‐skin outputs and inner joint angle at the same time. First, the rotation of joint 0 leads to the robot arm approaching palm, which reduces the proximity sensor output (Figure 5i, red curve). The robot arm will stop moving forward once the output drops below 16 pF, while moves forward again if the palm leaves. The temperature sensor output (voltage) will decrease (Figure 5i, pink curve), when the robot arm approaches to the heat gun. Once the voltage decreases below 1.6 V, the joint 0 rotates in reverse, while the joint 1 and joint 2 start to rotate away from the heat source simultaneously. The pressure sensor output (voltage) will increase (Figure 5i, blue curve) when the contact force is applied on the e‐skin. Once the voltage increases above 0.6 V, only the joint 0 rotates in reverse, while other joints keep unchanged. In addition, the humidity sensor output will decrease (Figure 5i, green curve) when the robot arm moves to high RH environment. Once the capacitance drops below 800 pF, the joints 0, 1, and 2 move away from the high RH environments. The above active safety control process indicates the specificity between the functional modules of the e‐skin system, and low‐coupling between the modules in the complicated environment upon the multifunctional e‐skin system. In addition, the cycle tests in various environmental conditions change for the active safety control of the robot are carried out. The drift rate of proximity, pressure, temperature, and humidity module are 3.65%, 4.72%, 1.86%, and 0.55% (Figure S23, Supporting Information), respectively. The use of commercial materials can improve the stability and reproducibility of the device, making the e‐skin system reasonably robust and reproducible in practice.

### 2.6 Improving Proximity Performance of the Multifunctional E‐Skin System via Machine Learning

**Figure 6:**
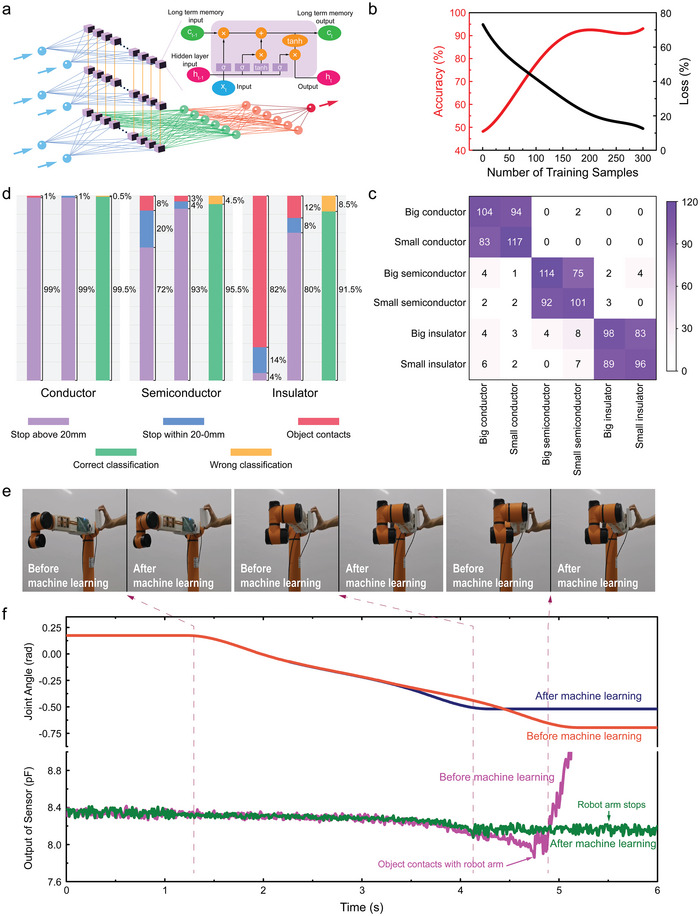
Improving proximity process performance of the multifunctional e‐skin system via machine learning. a) Schematic illustration of long short‐term (LSTM) memory network. b) The training results via LSTM network. c) Confusion matrix of the object conductivity and size in LSTM network. d) Comparison of the learning situation of different material objects. e) The snapshots of robot arm collaboration with nylon board before and after machine learning under the distance following control. f) The proximity sensor output and joint angle during the insulator approaching before and after machine learning under the distance following control.

During the active safety control, we found the robot arm cannot stop in front of insulating objects in time during the approaching (e.g., nylon and wood). This is because the insulator does not change the capacitance effectively as that of conductor, which induces that the proximity sensor output does not reach the threshold for the active safety control. To this end, a long short‐term memory network (LSTM, **Figure** **6**a) is developed to classify the conductivity of approaching objects. The capacitance value of the conductor, semiconductor, and insulator during approaching are used as input, while the object classification is taken as output (Figure 6a). In brief, the collected capacitance value sequence is input to the network input layer (blue spheres) first, and then the network starts to classify the object when the set threshold is reached. Following, the input sequence is processed and classified by the LSTM layer (purple blocks) and fully connected layers (green spheres). The LSTM unit includes Sigmod activation function, forget gate, input gate, cell state, and output gate (Supporting information). Finally, the classification is output through the Softmax layer (orange spheres), which is divided into six types with different sizes. The proximity sensing data for the objects with different conductivity are selected as the data set and accumulated in Table S2, Supporting Information. Among the data, 90% is employed to train the model, while other 10% is used for model validation. The accuracy gradually improves during the training process and reaches to 90.8% after 200 epochs of training (in Figure 6b). Meanwhile, the total loss gradually decreases to 12% after 300 rounds.

Figure 6c depicts the confusion matrix obtained by using the verification data set to verify the trained model. The designed LSTM network can classify the types of objects (conductor, semiconductor, and insulator) very well, with the accuracy rates of 99.5%, 95.5%, and 91.5% (Figure 6c), respectively. Figure 6d verifies the enhancement of the designed LSTM on the robot control performance in HRC. Two methods are used for robot arm control, one is to set the threshold uniformly and the other is to revise the threshold after LSTM classification. The robot approaches different objects at a constant speed from a specific position. For each type of the material, the first column is the experimental result before machine learning and the second column is the result after machine learning (Figure 6d). It is defined as “excellent” when the robot stops at 20 mm and beyond (Figure 6d, purple block), while it is defined as “feasible” when it stops in 0–20 mm (Figure 6d, blue block). Both stages can be taken as robot safety control. It is defined as “failure” when it collides the approaching object (Figure 6d red block), which is invalid in robot safety control. The probabilities are calculated to be 99%, 92%, and 18%, respectively, for the three types of materials that can achieve safe control before using machine learning. The probabilities increase to 100%, 97%, and 88% after machine learning. Impressively, it increases by 6.3 times for the insulator approaching after machine learning. The third column in Figure 6d is the running results upon the machine learning, in which the green part is the proportion of the ratio of successful classifications and the yellow part represents the proportion of the classification errors. The correct classification rates of the three materials are 99.5%, 95.5%, and 91.5%, respectively. Obviously, it is quite efficient for the robot safety control during the HRC via the proposed machine learning method.

Figure 6e shows snapshots of robot and human arms collaboration via the multifunctional e‐skin system before and after machine learning. Figure 6f compares the controlling performance of the manipulator as the insulator approaching under the two methods. The robot arm is also set to reciprocate from distal to proximal (Movie S4, Supporting Information). Normally, the capacitance change is so small to control the robot arm during the insulator approaching, which may induce to collide between the object and robot at a large probability. Upon the LSTM network, thresholds are optimized in real‐time. When it is judged that the external objects are conductors, semiconductors, and insulators, the thresholds change to 5.7%, 4.2%, and 2.7%, respectively. The threshold changes after machine learning so that the robot arm can stop in time when the nylon board approaches. Upon the machine learning, the robot arm equipped with multifunctional e‐skin can judge the conductivity of external objects in advance, which increases the intelligence and accuracy of the active safety control in HRC. Meanwhile, it provides a new carrier and platform for the interconnection of people, machines, and things in complicated environment.

## 3 Conclusion

In summary, we propose a multifunctional e‐skin via directly vertically integrated different sensing materials and structures together. The e‐skin exhibits multi‐information detection of the proximity distance, contact pressure, object temperature, and environmental relative humidity with the scope of 100–0 mm, 0–30 N, 20–120 °C, and 20–70%, respectively. The sensitivity of the four kinds sensors can be achieved to 0.72 mm^−1^, 16.34 N^−1^, 0.0032 °C^−1^, and 15.2 pF/%RH, respectively. The cross‐coupling errors are less than 1.96%, 1.08%, 2.65%, and 1.64%, respectively, after temperature compensation. Each single sensor exhibits linear or piecewise linear sensing properties with a low‐coupling feature. The cross‐coupling errors of the four kinds of information after temperature compensation are less than 1.96%, 1.08%, 2.65%, and 1.64%, respectively. For the reveal it validity, a commercial robot is accurately controlled via the multifunctional e‐skin system in the complicated environment. The following control and safety control exhibit both accuracy and high dynamic features. To improve the sensing performance to the insulating objects, machine learning has been employed to classify the conductivity during the object approaching, leading to set the threshold in dynamic. The accuracy for isolating the insulator increases from 18% to 88% after machine learning. In future, the multifunctional e‐skin system with low‐coupling will open an avenue for applications in human–machine collaboration and industrial safety control.

## 4 Experimental Section

### Materials

Carbon black (Ketjenblack EC‐600JD) was purchased from Lion Corporation (Tokyo, Japan). Nano‐SiO_2_ was purchased from Yuanjiang Chemical Co., Ltd (Shanghai, China). PDMS (Sylgard‐184) was purchased from Dow Corning (Midland, MI). Nickel target was purchased from Dream Material Co., Ltd (Beijing, China). PVDF powder, PVA granules, LiCl powder, *n*‐heptane, and DMSO solution were purchased from Aladdin Co., Ltd (Beijing, China). Comb‐shape FPC electrodes were ordered from Ruida Express Co., Ltd (Shenzhen, China). Acrylic molds were ordered from Jizhi Technology Co., Ltd (Qiqihar, China).

### Fabrication of the Temperature Sensitive Film

A 304 stainless steel sheet with a thickness of 0.3 mm was chosen as the reticle material. The serpentine pattern was cut with a wire EDM machine whose width was 400 µm and spacing was 300 µm, and the side length of the sensitive area was 11 mm. The fabricated metal mask was fixed on the surface of the clean PI film. 100 nm thick nickel pattern was fabricated on PI through magnetron sputtering (YWC‐450, Shenyang HUIKE). The substrate temperature was 25°C; the vacuum degree was 5 × 10^−4^ Pa; the argon gas pressure was 2.0 Pa. the sputtering power of the DC power supply was 125 W, in which time the sputtering rate was 0.2 Å s^−1^. Finally, the temperature sensitive layer was placed in a tube furnace (2F221236V, KJ GROUP) for annealing at 385 °C with 150 sccm argon for 60 min.

### Characterization of the Temperature Sensitive Film

Optical images were obtained using Digital Single Lens Reflex (D5600, Nikon). SEM images were taken with FESEM (SU8010, HITACHI). The heat source was provided by a heated magnetic stirrer (RCT digital, IKA). The temperature‐electrical properties of temperature sensitive layer were characterized by the LCR digital electric bridge (HG2810B, Huigao Electronic Co., Ltd.).

### Fabrication of the Humidity Sensitive Film

1.35 g PVDF powder, 0.15 g PVA granules, and 0.1 g LiCl powder were mixed with 13.5 g DMSO solution. The mixed solution was stirred at 80°C for 8 h on a heated magnetic stirrer (RCT digital, IKA), and then placed in a vacuum drying oven for 6 h to completely remove air bubbles. Then the mixed solution was poured on a glass sheet with a thickness of 1 mm. Finally, the glass sheets were put into the oven to cure at 80 °C for 4 h to get humidity sensitive layer. The thickness of the humidity sensitive material was 80 µm. The diagram of the corresponding fabrication process is shown in Figure S8, Supporting Information.

### Characterization of the Humidity Sensitive Film

Optical images were obtained using Digital Single Lens Reflex (D5600, Nikon). SEM images were taken with FESEM (SU8010, HITACHI). The humidity source was provided by home‐made humidity generating device shown in Figure S17, Supporting Information. The humidity‐electrical properties of temperature sensitive layer were characterized by the LCR digital electric bridge (HG2810B, Huigao Electronic Co., Ltd.).

### Fabrication of the Multifunctional E‐Skin

Flexible substrates and sealing layer were fabricated by PDMS with specific acrylic molds. The composite flexible films were made according to ref. [55]. The bottom comb‐shape FPC electrodes were adhered to the flexible substrate by RTV room temperature cured silicone rubber. The top comb‐shape FPC electrodes were adhered to the sealing layer by RTV room temperature cured silicone rubber. The sealing layer, composite flexible films, and flexible substrate were bonded together by surface plasma treatment. Flexible substrates, sealing layer, composite flexible films, humidity sensitive film, and bottom and top comb‐shape FPC electrodes are shown in Figure S1, Supporting Information.

### Static Measurements of the Multifunctional E‐Skin

Optical images were obtained using Digital Single Lens Reflex (D5600, Nikon). The mechanical test was carried out with a measurement system containing a motorized test stand connected with a force gauge (ESM1500G, Mark‐10). For the measurement, the object was fixed on the motorized test stand and electrically grounded. The proximity distance was realized via the movement of the motorized test stand. The contact force on the sensor can be read out from the force gauge in real time. The humidity source was provided by the humidity generating device, including compressed air through a water conical flask, pure nitrogen, a flow meter (D07‐19F, Beijing Sevenstar Electronics Co. Ltd.), and a gas mixing chamber. Controllable regulation of humidity was achieved by regulating the flow through the flow meter. The heat source was provided by the heating plate (JRD‐500S, KINGBALI). Temperature regulation can be achieved through changing the voltage across the heating plate. The electrical properties of multi‐information sensor units were characterized by the LCR digital electric bridge (HG2810B, Huigao Electronic Co., Ltd.). The sensor array was connected to a home‐made signal process setup to collect static electrical signals.

### Principle and Performance of the Home‐Made Capacitance–Resistance Data Acquisition Circuit

The mutual capacitance was measured by FDC2214, which was calculated by measuring the oscillation frequency of the LC resonator. The current in the sensor was amplified by a current amplifier MCP6004T, and the digital quantity was transmitted to the lower computer after A/D conversion. A STM32 MCU was chosen as the lower computer, which communicates with the upper computer through the RS232 serial port. It can achieve capacitance measurement from 0.01 pF to 10 nF and resistance measurement from 1kΩ to 1MΩ. The self‐made capacitance–resistance data acquisition circuit contains MCU circuit, capacitance test circuit, resistance test circuit, serial communication, FPC interface, power interface, and voltage regulator circuit module.

### Control Principle of the Collaborative Robot Arms

The robot arm control was tested on a collaborative robot (i10, AUBO Robot Co., Ltd.). Different ambient humidity was provided by commercial humidifier (SC03L‐80, Supor Co., Ltd.). Different environmental temperature was provided by a heat gun (A‐2000TS, Anritson Co., Ltd.). Palm pressing provides contact force and palm blocking was regarded as an object approaching. The robot arm was set to reciprocate from distal to proximal. The distance and pressure following control was similar to damping control. The forward movement speed of the robot arm was controlled based on the distance between the palm and the robot inverse proportionally. Similarly, the movement speed in reverse was controlled based on the contact pressure proportionally. The active safety control of the manipulator was similar to the interrupt control commonly utilized in kinematic control. When the distance, pressure, humidity, or temperature detected by the sensor reached the set value, the motion of the robot arm changed. After reaching the target position, the active safety control or following control cycle ends.

### Dynamic Measurements of the Multifunctional E‐Skin

The multi‐information sensing systems were integrated into the collaborative robot. The robot will be able to feel the change of proximity distance, contact pressure, environment humidity, and object temperature, and then complete the corresponding control actions according to the changes. The network cable was used to connect the control cabinet of the real manipulator to the computer, and used Robot Operating System (ROS) to control the motion of the manipulator. For the calculation of the robot trajectory, the C++ interface move_group_interface in Moveit!, the GUI of RViz, and other interfaces were used to complete the pairing robot path planning, path execution, inverse kinematics, and other functions. In addition to the native functions of ROS, the SDK interface of the robot arm was also used to send the trajectory path calculated by Moveit! to the robot arm in real time through the transparent transmission interface, so as to complete the real‐time control of the robot arm trajectory by ROS.

## Conflict of Interest

The authors declare no conflict of interest.

## Supporting information

Supporting Information

Supplemental Movie 1

Supplemental Movie 2

Supplemental Movie 3

Supplemental Movie 4

## Data Availability

The data that support the findings of this study are available from the corresponding author upon reasonable request.

## References

[1] C. P. Day , Engineering 2018, 4, 440.

[2] J. Zhou , P. Li , Y. Zhou , B. Wang , J. Zang , L. Meng , Engineering 2018, 4, 11.

[3] M. P. Pacaux‐Lemoine , D. Trentesaux , G. Z. Rey , P. Millot , Comput. Ind. Eng. 2017, 111, 581.

[4] P. Wang , H. Liu , L. Wang , R. Gao , CIRP Ann. Manuf. Technol. 2018, 67, 17.

[5] T. N. Do , Y. Visell , Sci. Rep. 2017, 7, 1753. 28496101 10.1038/s41598-017-01898-8 PMC5431990

[6] T. Patino , R. Mestre , S. Sanchez , Lab Chip 2016, 16, 3626. 27550016 10.1039/c6lc90088g

[7] J. R. Ruiz‐Sarmiento , C. Galindo , J. Gonzalez‐Jimenez , Int. J. Rob. Res. 2017, 36, 131.

[8] S. Cosar , M. Fernandez‐Carmona , R. Agrigoroaie , J. Pages , F. Ferland , F. Zhao , S. Yue , N. Bellotto , A. Tapus , Int. J. Social Rob. 2020, 12, 779.

[9] Y. Pang , K. Zhang , Z. Yang , S. Jiang , Z. Ju , Y. Li , X. Wang , D. Wang , M. Jian , Y. Zhang , R. Liang , H. Tian , Y. Yang , T. L. Ren , ACS Nano 2018, 12, 2346. 29378401 10.1021/acsnano.7b07613

[10] T. Yang , D. Xie , Z. Li , H. Zhu , Mater. Sci. Eng.: R: Rep. 2017, 115, 1.

[11] K. Xu , Y. Lu , K. Takei , Adv. Mater. 2019, 4, 1800628.

[12] Y. Kim , A. Chortos , W. Xu , Y. Liu , J. Y. Oh , D. Son , J. Kang , A. M. Foudeh , C. Zhu , Y. Lee , S. Niu , J. Liu , R. Pfattner , Z. Bao , T.‐W. Lee , Science 2018, 360, 998. 29853682 10.1126/science.aao0098

[13] Q. Hua , J. Sun , H. Liu , R. Bao , R. Yu , J. Zhai , C. Pan , Z. L. Wang , Nat. Commun. 2018, 9, 244. 29339793 10.1038/s41467-017-02685-9 PMC5770430

[14] S. Sundaram , P. Kellnhofer , Y. Li , J.‐Y. Zhu , A. Torralba , W. Matusik , Nature 2019, 569, 698. 31142856 10.1038/s41586-019-1234-z

[15] W.‐D. Wu , K. Ke , J. Jia , J.‐H. Pu , X. Zhao , R.‐Y. Bao , Z.‐Y. Liu , L. Bai , K. Zhang , M.‐B. Yang , W. Yang , Small 2018, 18, 2103734. 10.1002/smll.202103734 34825473

[16] Y. Chen , Y. Sun , Y. Wei , J. Qiu , Adv. Mater. Technol. 2023, 8, 2201352.

[17] J. C. Yang , J. Mun , S. Y. Kwon , S. Park , Z. Bao , S. Park , Adv. Mater. 2019, 31, 1904765. 10.1002/adma.201904765 31538370

[18] B. Yang , L. Jiang , J. Bionic Eng. 2023, 20, 267.

[19] Z. Lei , P. Wu , Nat. Commun. 2018, 9, 1134. 29555905 10.1038/s41467-018-03456-w PMC5859265

[20] Z. Zou , C. Zhu , Y. Li , X. Lei , W. Zhang , J. Xiao , Sci. Adv. 2018, 4, eaaq0508. 29487912 10.1126/sciadv.aaq0508 PMC5817920

[21] Y. Lee , J. Park , A. Choe , S. Cho , J. Kim , H. Ko , Adv. Funct. Mater. 2020, 30, 1904523.

[22] H. Liu , Q. Li , S. Zhang , R. Yin , X. Liu , Y. He , K. Dai , C. Shan , J. Guo , C. Liu , C. Shen , X. Wang , N. Wang , Z. Wang , R. Wei , Z. Guo , J. Mater. Chem. 2018, 6, 12121.

[23] C. M. Boutry , M. Negre , M. Jorda , O. Vardoulis , A. Chortos , O. Khatib , Z. Bao , Sci. Rob. 2018, 3, eaau6914. 10.1126/scirobotics.aau6914 33141713

[24] I. You , D. G. Mackanic , N. Matsuhisa , J. Kang , J. Kwon , L. Beker , J. Mun , W. Suh , T. Y. Kim , J. B.‐H. Tok , Z. Bao , U. Jeong , Science 2020, 370, 961. 33214277 10.1126/science.aba5132

[25] B. W. An , S. Heo , S. Ji , F. Bien , J.‐U. Park , Nat. Commun. 2018, 9, 2458. 29970893 10.1038/s41467-018-04906-1 PMC6030134

[26] D. H. Ho , Q. Sun , S. Y. Kim , J. T. Han , D. H. Kim , J. H. Cho , Adv. Mater. 2016, 28, 2601. 26833961 10.1002/adma.201505739

[27] W. Liu , N. Liu , Y. Yue , J. Rao , F. Cheng , J. Su , Z. Liu , Y. Gao , Small 2018, 14, 1704149. 10.1002/smll.201704149 29527801

[28] B. Neji , N. Ferko , R. Ghandour , A. S. Karar , H. Arbess , Sensors 2021, 21, 318. 33466505 10.3390/s21010318 PMC7796505

[29] C.‐Y. Lee , C.‐H. Lin , Y.‐M. Lo , Sensors 2011, 11, 3706. 22163817 10.3390/s110403706 PMC3231302

[30] A. Davidson , A. Buis , I. Glesk , IEEE Sens. J. 2017, 17, 6682.

[31] G. Lee , J. H. Son , S. Lee , S. W. Kim , D. Kim , N. N. Nguyen , S. G. Lee , K. Cho , Adv. Sci. 2021, 8, 2002606. 10.1002/advs.202002606 PMC8097346 33977042

[32] H. Guo , X. Pu , J. Chen , Y. Meng , M.‐H. Yeh , G. Liu , Q. Tang , B. Chen , D. Liu , S. Qi , C. Wu , C. Hu , J. Wang , Z. L. Wang , Sci. Rob. 2018, 3, eaat2516. 10.1126/scirobotics.aat2516 33141730

[33] K. Dong , Z. Wu , J. Deng , A. C. Wang , H. Zou , C. Chen , D. Hu , B. Gu , B. Sun , Z. L. Wang , Adv. Mater. 2018, 30, 1804944. 10.1002/adma.201804944 30256476

[34] J. H. Lee , J. S. Heo , Y.‐J. Kim , J. Eom , H. J. Jung , J.‐W. Kim , I. Kim , H.‐H. Park , H. S. Mo , Y.‐H. Kim , Adv. Mater. 2020, 32, 2000969. 10.1002/adma.202000969 32310332

[35] Q. Zhang , Q. Liang , Z. Zhang , Z. Kang , Q. Liao , Y. Ding , M. Ma , F. Gao , X. Zhao , Y. Zhang , Adv. Funct. Mater. 2018, 28, 1703801.

[36] S. Xie , Y. Zhang , M. Jin , C. Li , Q. Meng , IEEE Sens. J. 2020, 21, 2757.

[37] B. Ward‐Cherrier , N. Pestell , L. Cramphorn , B. Winstone , M. E. Giannaccini , J. Rossiter , N. F. Lepora , Soft Robot 2018, 5, 216. 29297773 10.1089/soro.2017.0052 PMC5905869

[38] L. Zhang , J. Pan , Z. Zhang , H. Wu , N. Yao , D. Cai , Y. Xu , J. Zhang , G. Sun , L. Wang , W. Geng , W. Jin , W. Fang , D. Di , L. Tong , Opto‐Electron. Adv. 2020, 3, 190022.

[39] C.‐Y. Tang , X. Zhao , J. Jia , S. Wang , X.‐J. Zha , B. Yin , K. Ke , R.‐Y. Bao , Z.‐Y. Liu , Y. Wang , K. Zhang , M.‐B. Yang , W. Yang , Nano Energy 2021, 90, 106603.

[40] H. Kong , Z. Song , W. Li , M. Chen , Y. Bao , Z. Liu , D. Qu , Y. Ma , Z. Wang , D. Han , L. Niu , Nano Energy 2022, 100, 107498.

[41] D.‐I. Kim , T. Q. Trung , B.‐U. Hwang , J.‐S. Kim , S. Jeon , J. Bae , J.‐J. Park , N.‐E. Lee , Sci. Rep. 2015, 5, 12705. 26223845 10.1038/srep12705 PMC4520005

[42] F. Zhang , Y. Zang , D. Huang , C. Di , D. Zhu , Nat. Commun. 2015, 6, 8356. 26387591 10.1038/ncomms9356 PMC4595753

[43] M. Zhu , Q. Shi , T. He , Z. Yi , Y. Ma , B. Yang , T. Chen , C. Lee , ACS Nano 2019, 13, 1940. 30741521 10.1021/acsnano.8b08329

[44] S. Wang , F. Gao , Y. Hu , S. Zhang , H. Shang , C. Ge , B. Tan , X. Zhang , J. Zhang , P. A. Hu , Chem. Eng. J. 2022, 443, 136446.

[45] G. Li , S. Liu , L. Wang , R. Zhu , Sci. Rob. 2020, 5, eabc8134. 10.1126/scirobotics.abc8134 33328298

[46] T. Someya , Y. Kato , T. Sekitani , S. Iba , Y. Noguchi , Y. Murase , H. Kawaguchi , T. Sakurai , Proc. Natl. Acad. Sci. U. S. A. 2005, 102, 12321. 16107541 10.1073/pnas.0502392102 PMC1187825

[47] J. Oh , J. C. Yang , J.‐O. Kim , H. Park , S. Y. Kwon , S. Lee , J. Y. Sim , H. W. Oh , J. Kim , S. Park , ACS Nano 2018, 12, 7546. 29995382 10.1021/acsnano.8b03488

[48] X. Wu , J. Zhu , J. W. Evans , C. Lu , A. C. Arias , Adv. Funct. Mater. 2021, 31, 2010824.

[49] J. Park , M. Kim , Y. Lee , H. S. Lee , H. Ko , Sci. Adv. 2015, 1, e1500661. 26601303 10.1126/sciadv.1500661 PMC4646817

[50] J. W. Gong , Q. F. Chen , M. R. Lian , N. C. Liu , C. Daoust , IEEE Sens. J. 2006, 1, 139.

[51] Y. Yang , Z.‐H. Lin , T. Hou , F. Zhang , Z. L. Wang , Nano Res. 2012, 5, 888.

[52] K. Agarwal , V. Kaushik , D. Varandani , A. Dhar , B. R. Mehta , J. Alloys Compd. 2016, 681, 394.

[53] E. M. F. Vieira , J. Figueira , A. L. Pires , J. Grilo , M. F. Silva , A. M. Pereira , L. M. Goncalves , J. Alloys Compd. 2019, 774, 1102.

[54] T. Yamada , Y. Hayamizu , Y. Yamamoto , Y. Yomogida , A. Izadi‐Najafabadi , D. N. Futaba , K. Hata , Nat. Nanotechnol. 2011, 6, 296. 21441912 10.1038/nnano.2011.36

[55] S. Xu , Z. Fan , S. Yang , Y. Zhao , L. Pan , Chem. Eng. J. 2021, 404, 126064.

[56] J.‐H. Kong , N.‐S. Jang , S.‐H. Kim , J.‐M. Kim , Carbon 2014, 77, 199.

[57] N. Lu , C. Lu , S. Yang , J. Rogers , Adv. Funct. Mater. 2012, 22, 4044.

[58] M. Li , T. Wang , Y. Zhang , H. Zhou , Z. Huang , D. Li , J. Mater. Chem. 2018, 6, 5877.

[59] C. Ge , Z. Wang , Z. Liu , T. Wu , S. Wang , X. Ren , D. Chen , J. Zhao , P.‐A. Hu , J. Zhang , Adv. Intell. Syst. 2022, 4, 2100213.

[60] H. Guan , R. Yang , W. Li , Y. Tao , C. Chen , H. Tai , Y. Su , Y. Wang , Y. Jiang , W. Li , Sens. Actuators, B 2023, 377, 132996.

[61] E. Ganbold , E. S. Kim , Y. Li , F. Yin , P. K. Sharma , J.‐B. Jeon , J.‐M. Oh , D. N. Lee , N. Y. Kim , ACS Appl. Mater. Interfaces 2023, 15, 4559. 36633438 10.1021/acsami.2c20499

[62] C. Ge , Z. Duan , R. Li , H. Chen , T. Li , P.‐A. Hu , Z. Wang , J. Zhao , J. Zhang , Sens. Actuators, A 2022, 343, 113676.

