# Supplementary material for: Integrated Multifunctional Electronic Skins with Low‐Coupling for Complicated and Accurate Human–Robot Collaboration
Source: Adv Sci (Weinh). 2023 May 17;10(20):2301341. doi: 10.1002/advs.202301341 (PMC10369299; doi:10.1002/advs.202301341)
Supplement: Supplementary file 1 — Supporting Information [file ADVS-10-2301341-s004.pdf]

ADVANCED SCIENCE
Open Access

Supporting Information

for *Adv. Sci.*, DOI 10.1002/advs.202301341

Integrated Multifunctional Electronic Skins with Low-Coupling for Complicated and Accurate Human–Robot Collaboration

Chuanyang Ge, Xuyang An, Xinxin He, Zhan Duan, Jiatai Chen, PingAn Hu, Jie Zhao, Zhenlong Wang and Jia Zhang**

Supporting Information

Integrated multifunctional electronic skins with low-coupling for complicated and accurate human-robot collaboration

Chuanyang Ge[#], Xuyang An[#], Xinxin He, Zhan Duan, Jiatai Chen, PingAn Hu, Jie Zhao, Zhenlong Wang[*] and Jia Zhang[*]

C. Ge, X. An, X. He, Z. Duan, J. Chen, Prof. J. Zhao, Prof. Z. Wang, Prof. J. Zhang
State Key Laboratory of Robotics and System,
Harbin Institute of Technology,
Harbin 150080, China.
E-mail: zhangjia@hit.edu.cn; wangzl@hit.edu.cn

Prof. P. Hu,
Key Laboratory of Microsystems and Microstructure Manufacturing, Ministry of Education,
Harbin Institute of Technology,
Harbin 150080, China.

Algorithm of long-short term memory (LSTM)

The core concept of LSTM is cell state and "gate" structure. The cell state is equivalent to the path of information transmission, and the cell state can transmit relevant information in the process of sequence processing all the time. The LSTM unit includes Sigmod activation function, forget gate, input gate, cell state and output gate. The gate structure contains the sigmoid activation function. The Sigmoid activation function is similar to the tanh function, the difference is that sigmoid compresses the value between 0 and 1.

The function of the forget gate is to decide which information should be discarded or kept. The information from the previous hidden state and the current input information are passed to the sigmoid function at the same time, and the output value is between 0 and 1. The closer to 0 means that it should be discarded, and the closer to 1 means that it should be retained. The forget gate can be calculated as

$$f_t = \sigma(W_f \cdot [h_{t-1}, x_t] + b_f) \quad \text{(S1)}$$

The input gate is used to update the cell state. First, the information of the hidden state of the previous layer and the information of the current input are passed to the sigmoid function. Adjust the value between 0~1 to decide which information to update. 0 means not important, 1 means important. Secondly, the information of the hidden state of the previous layer and the information of the current input must be passed to the tanh function to create a new vector of candidate values. Finally, the output value of sigmoid is multiplied by the output value of tanh, and the output value of sigmoid will determine which information in the output value of tanh is important and needs to be preserved. The input gate can be calculated as

$$i_t = \sigma(W_i \cdot [h_{t-1}, x_t] + b_i) \quad \text{(S2)}$$

$$\tilde{C}_t = \tanh(W_C \cdot [h_{t-1}, x_t] + b_C) \quad \text{(S3)}$$

First, the cell state of the previous layer is multiplied point by point by the forgetting vector. If it is multiplied by a value close to 0, it means that in the new cell state, this information needs to be discarded. Then add this value to the output value of the input gate point by point, and update the new information discovered by the neural network to the cell state. At this point, the updated cell state is obtained. The cell state can be calculated as

$$C_t = f_t * C_{t-1} + i_t * \tilde{C}_t \quad \text{(S4)}$$

The output gate is used to determine the value of the next hidden state, which contains the information of the previous input. First, we pass the previous hidden state and the current input into the sigmoid function, and then pass the newly obtained cell state to the tanh function. Finally, the output of tanh is multiplied by the output of sigmoid to determine what information the hidden state should carry. Then use the hidden state as the output of the current cell, and pass the new cell state and new hidden state to the next time step. The output gate can be calculated as

$$o_t = \sigma(W_o \cdot [h_{t-1}, x_t] + b_o) \quad \text{(S5)}$$

$$h_t = o_t * \tanh(C_t) \quad \text{(S6)}$$

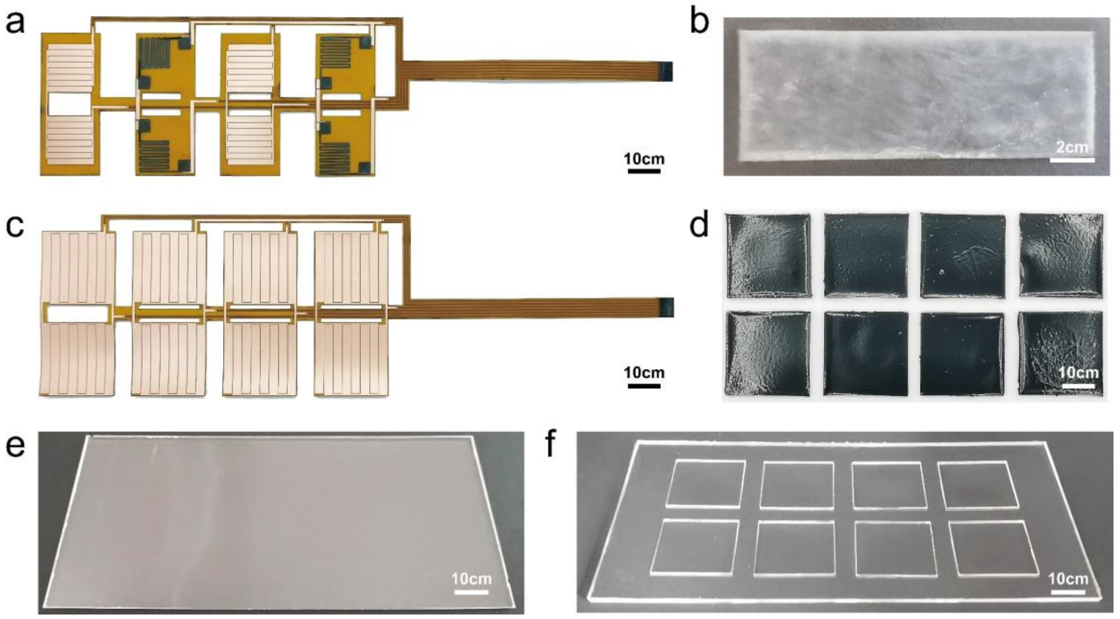

Figure S1. Optical images of the parts of the multifunctional e-skin. (a) Top coplanar electrode with 100nm-thick nickel film. (b) Humidity sensitive film. (c) Bottom coplanar electrode. (d) Flexible composite films formed by carbon black fillers in the polydimethylsiloxane (PDMS) matrix. (e) Silicone flexible substrate. (f) Silicone elastomer sealing layer.

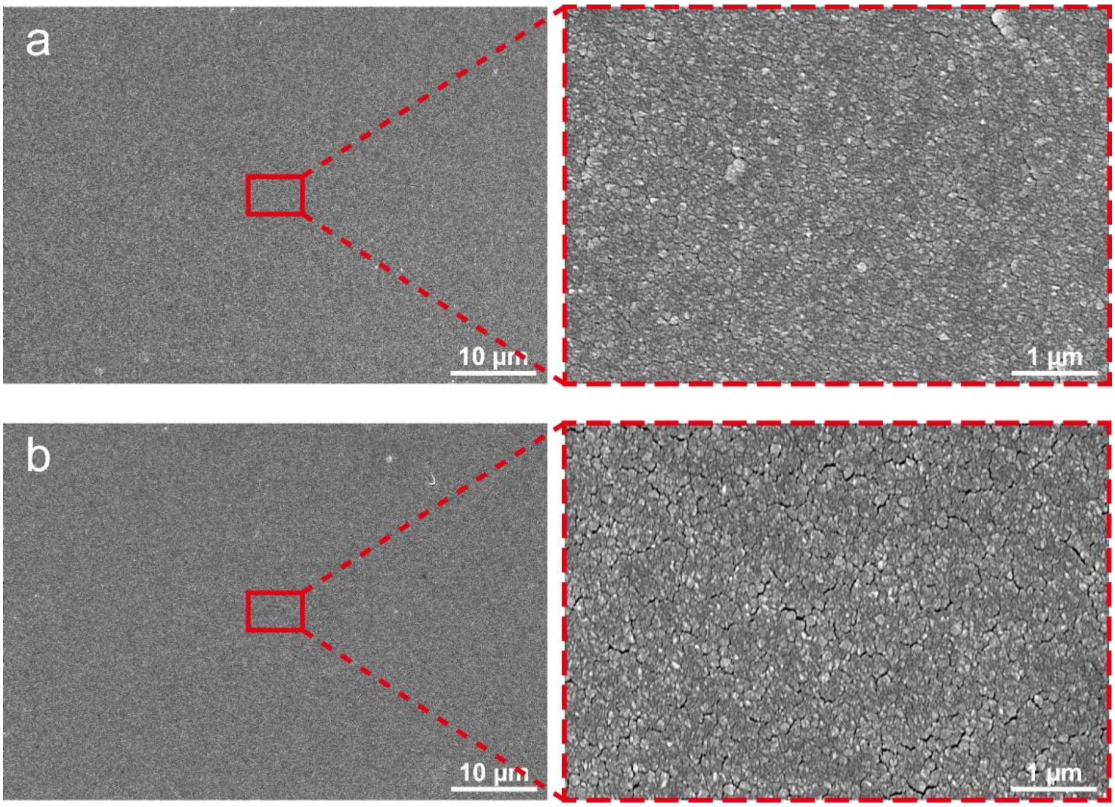

Figure S2. SEM images of the temperature sensitive film. (a) Before annealing. (b) After annealing.

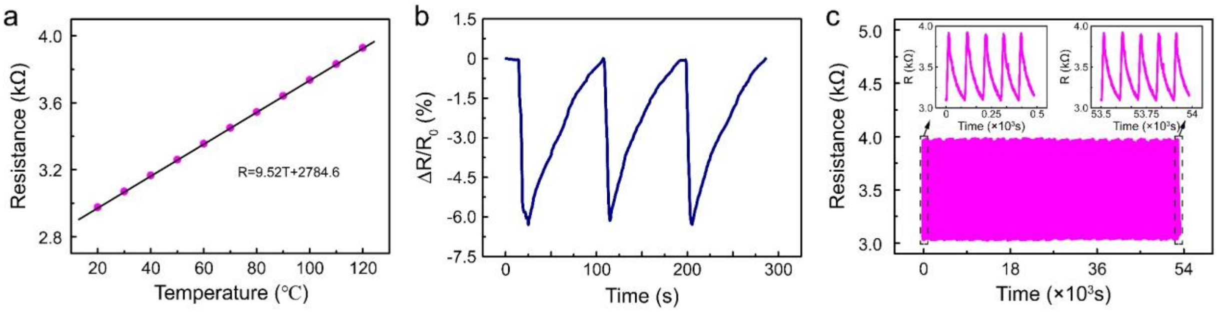

Figure S3. Properties of the temperature sensitive layer. (a) Resistance of the temperature sensitive layer with respect to the temperature. (b) The continuous response and recovery experiments at room temperature (20℃) and 0℃. (c) The resistance changes with the temperature fluctuating at 30℃ and 120℃ repeatedly.

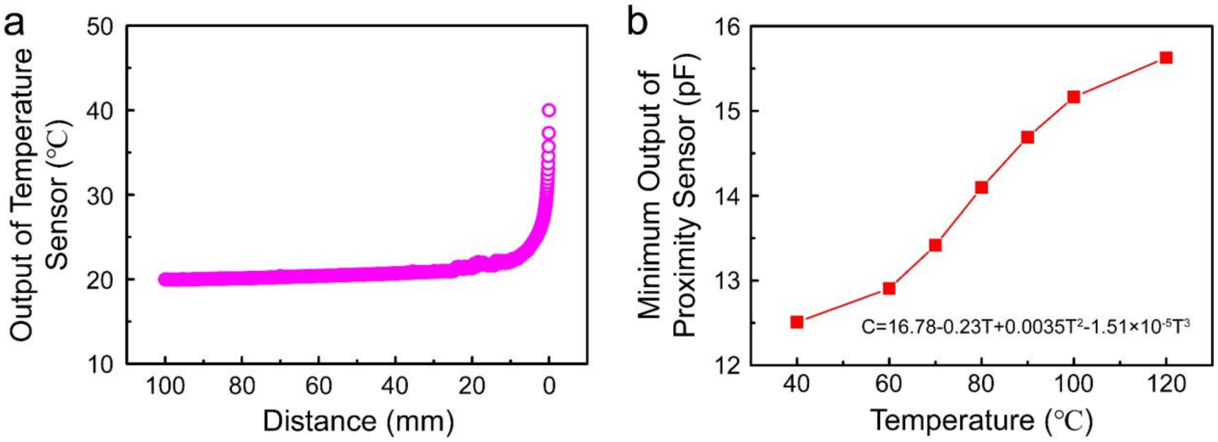

Figure S4. Properties of the temperature/proximity/pressure sensor unit as object approaching. (a) The output of the temperature sensor when the object under 40℃ approaches from 100mm to 0mm. (b) The minimum output of the proximity sensor with the temperature range from 40℃ to 120℃.

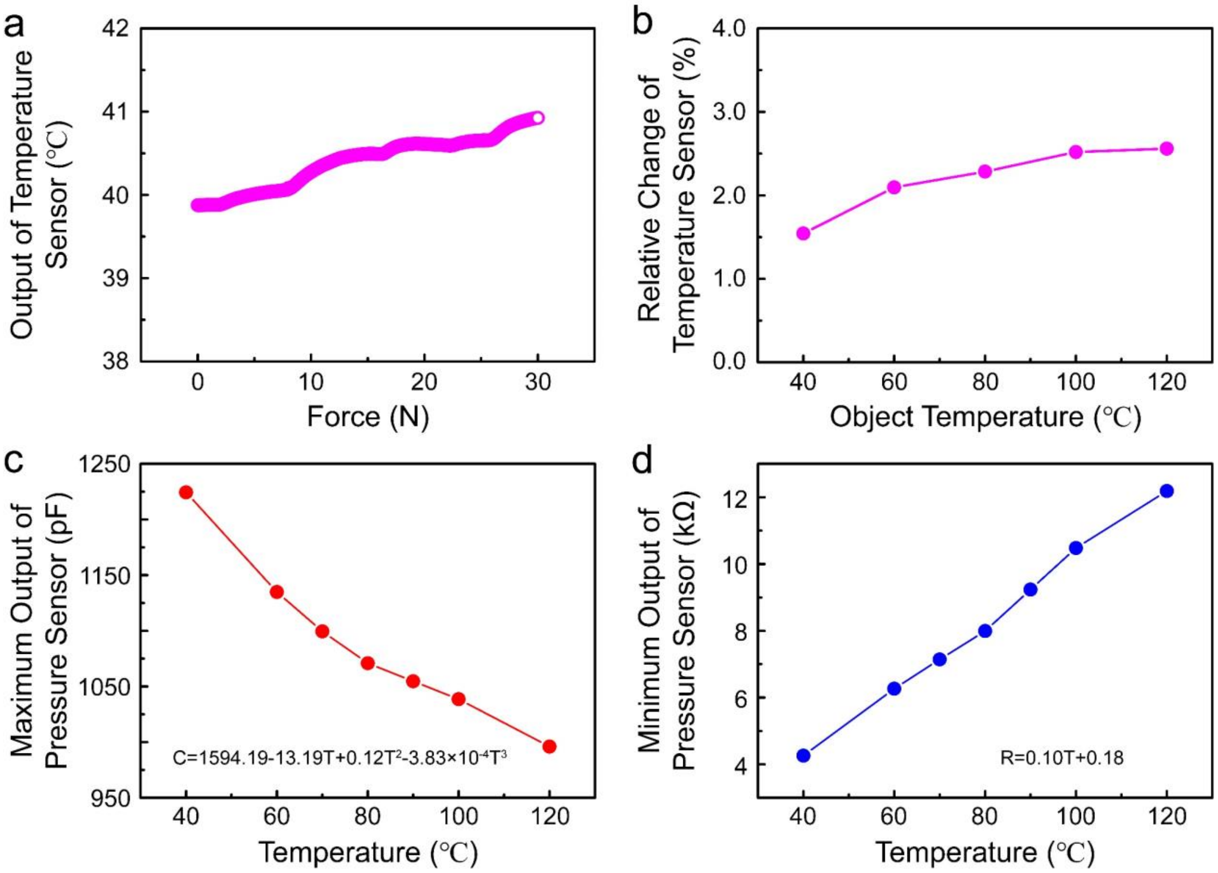

Figure S5. Properties of the temperature/proximity/pressure sensor unit as object contacts. (a) The output of the temperature sensor at 40℃ when the contact pressure is from 0N to 30N. (b) Relative change of the temperature sensor at 30N with the temperature from 40℃ to 120℃. (c) The maximum output of the pressure sensor in capacitive mode with the temperature range from 40℃ to 120℃ at 30N. (d) The minimum output of the pressure sensor in resistive mode with the temperature range from 40℃ to 120℃ at 30N.

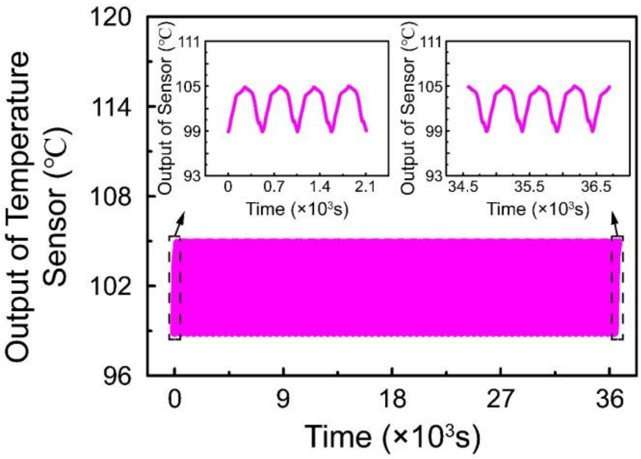

Figure S6. The variation of resistance of temperautre sensor at 100°C with repeated pressure fluctuations at 0 and 30N.

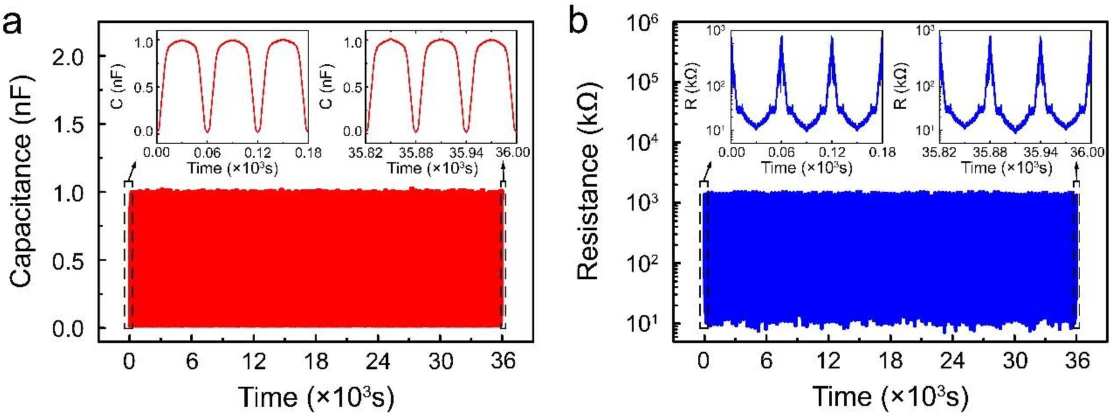

Figure S7. The variation of capacitance and resistance of pressure sensor at 120°C with repeated pressure fluctuations at 0 and 30N.

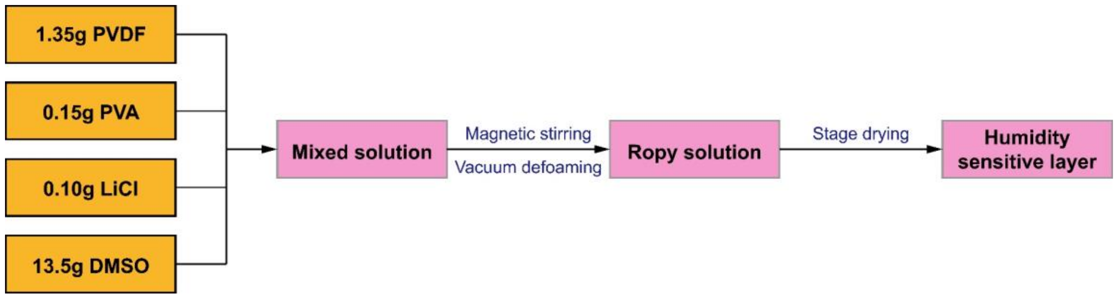

Figure S8. Fabrication steps of humidity sensitive films.

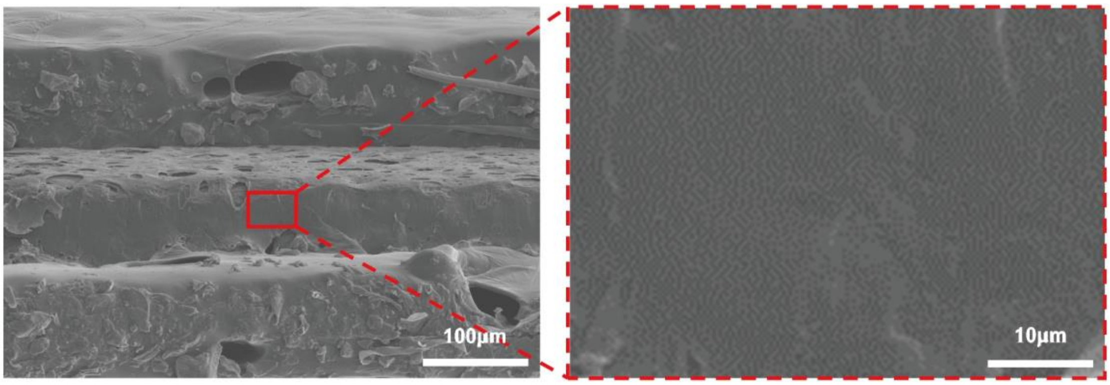

Figure S9. SEM image of cross section of the humidity sensitive film.

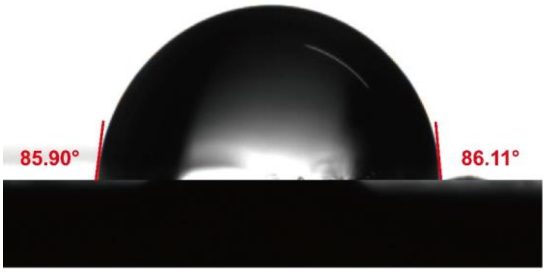

Figure S10. The water contact angle of the PVDF/PVA/LiCl humidity sensitive film.

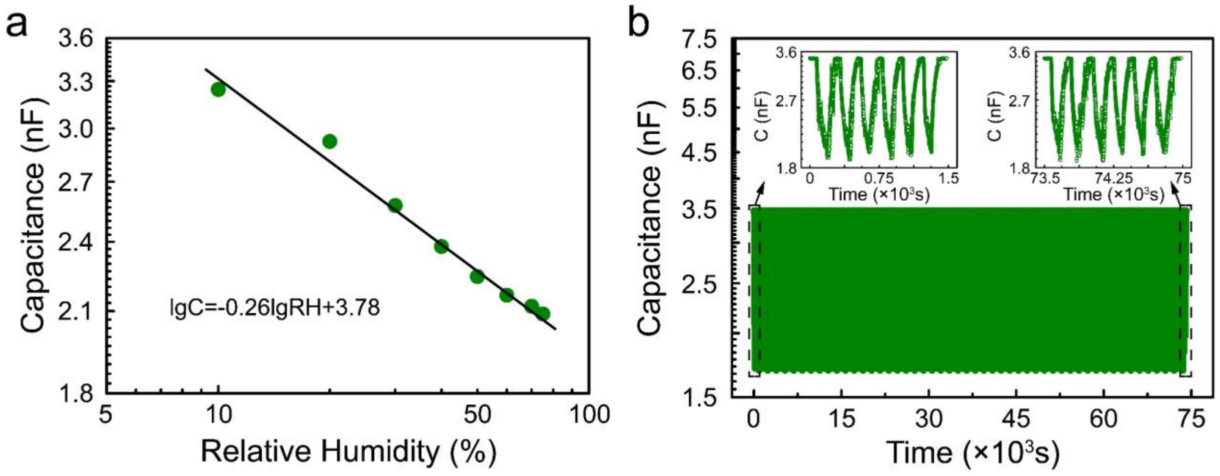

Figure S11. Properties of the humidity sensitive layer. (a) Capacitance of the humidity sensitive layer with respect to the relative humidity. (b) The capacitance changes with the humidity fluctuating at 0 and 70% repeatedly.

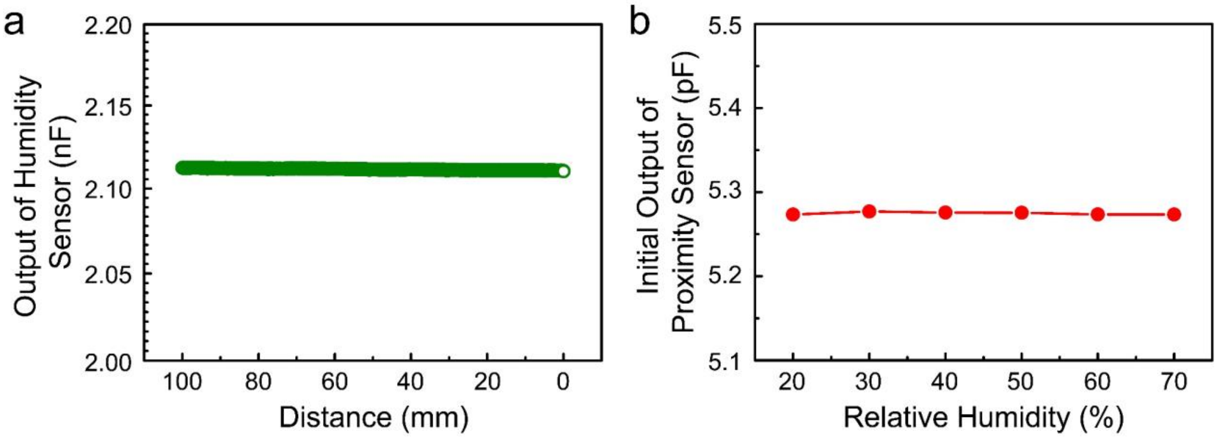

Figure S12. Properties of the humidity/proximity/pressure sensor unit as object approaching. (a) The output of the humidity sensor when the object approaches from 100mm to 0mm with the environment humidity of 50%. (b) The initial output of the proximity sensor with the relative humidity range from 20% to 70%.

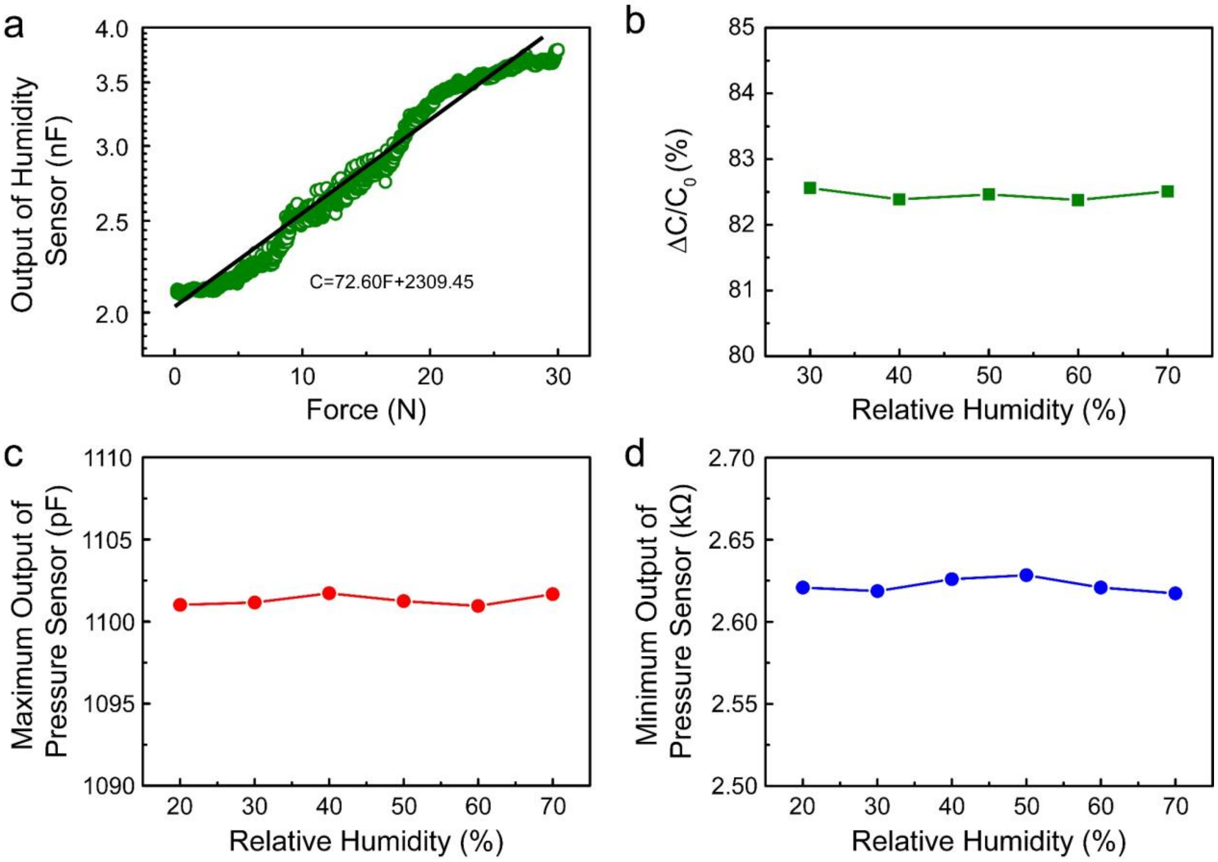

Figure S13. Properties of the humidity/proximity/pressure sensor unit as object contact. (a) The output of the humidity sensor at 50% when the contact pressure is from 0N to 30N. (b) Relative change of the humidity sensor at 30N with the relative humidity from 30% to 70%. (c) The maximum output of the pressure sensor in capacitive mode with the humidity range from 20% to 70% at 30N. (d) The minimum output of the pressure sensor in resistive mode with the temperature range from 20% to 70% at 30N.

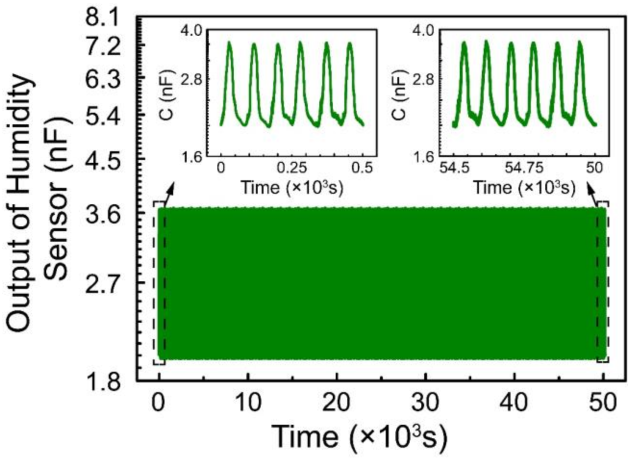

Figure S14. The variation of capacitance of humidity sensor at 70% with repeated pressure fluctuations at 0 and 30N.

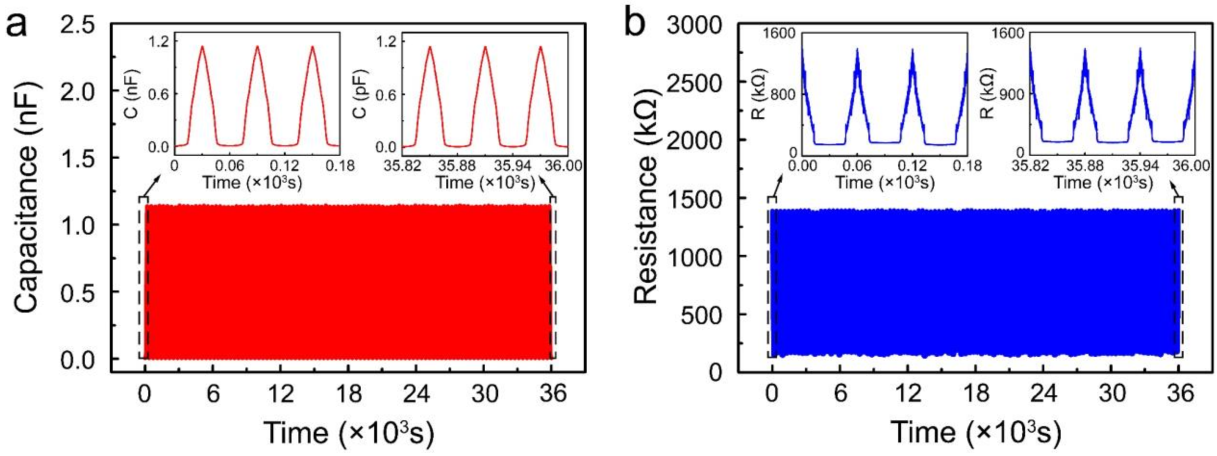

Figure S15. The variation of capacitance and resistance of pressure sensor at 70% with repeated pressure fluctuations at 0 and 30N.

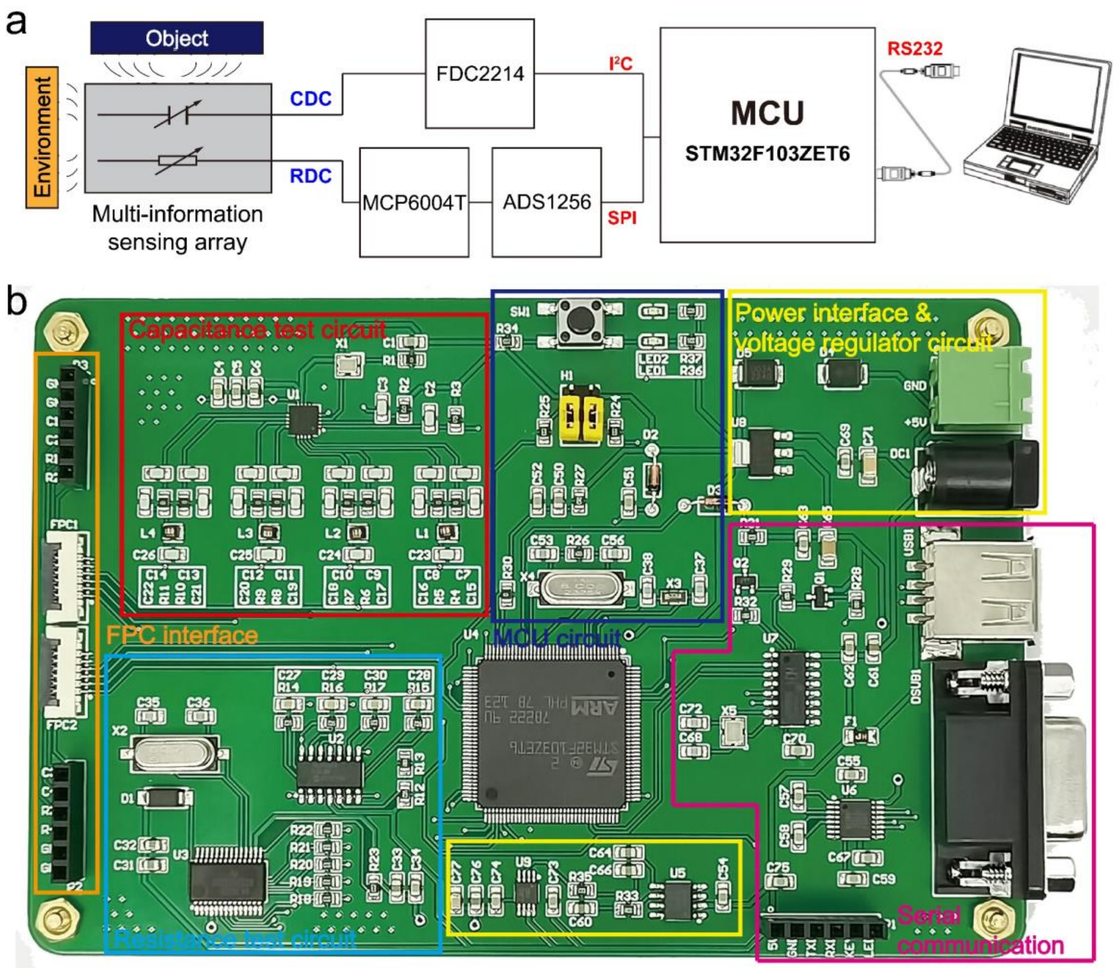

Figure S16. Capacitance-resistance data acquisition circuit. (a) Schematic diagram of data acquisition circuit system. (b) PCB image figure of capacitor-resistance data acquisition circuit.

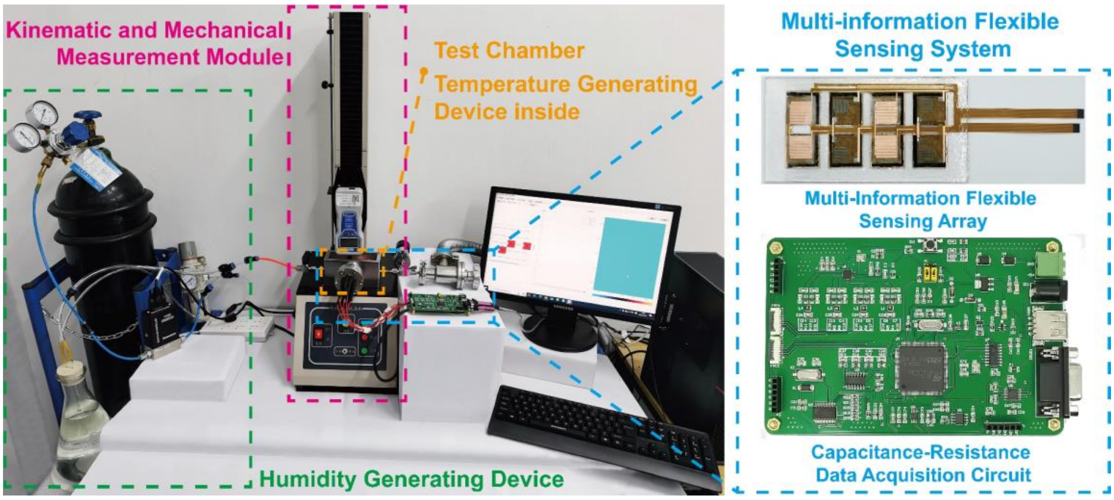

Figure S17. Multifunctional e-skin static performance experimental platform.

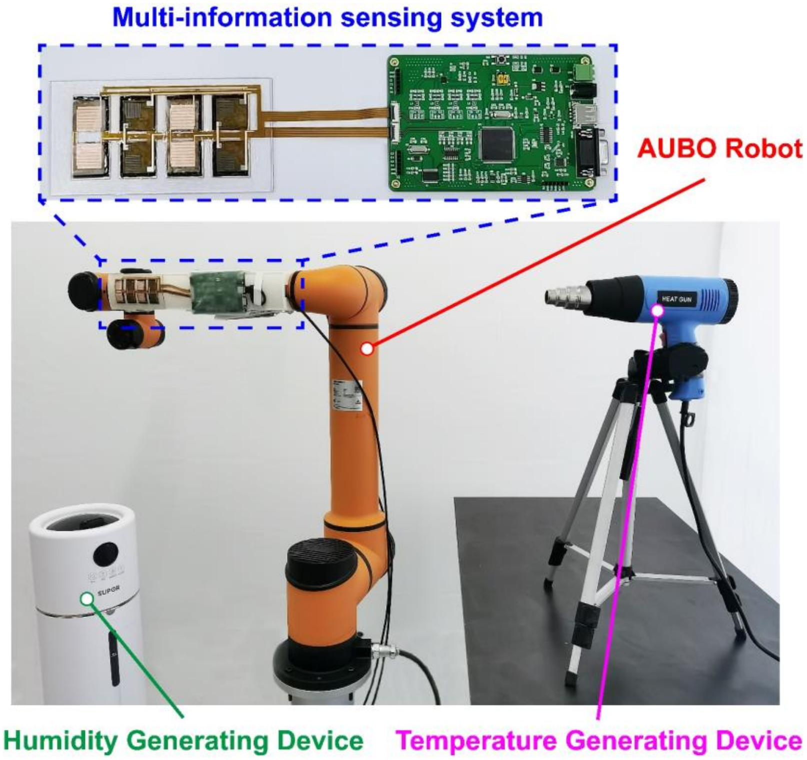

Figure S18. Multifunctional e-skin systems application platform.

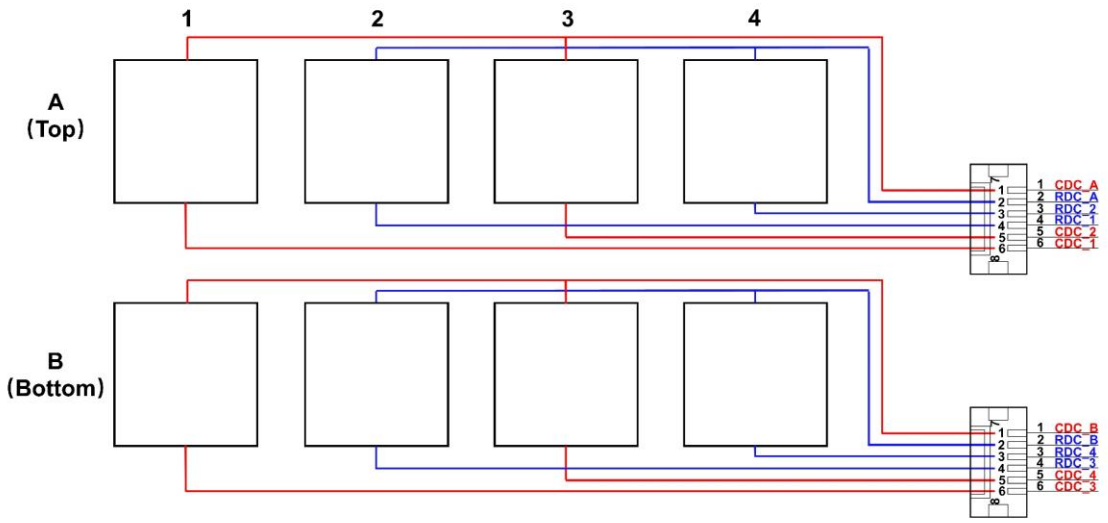

Figure S19. Sensor array measurement circuit capacitance-resistance input matrix interface.

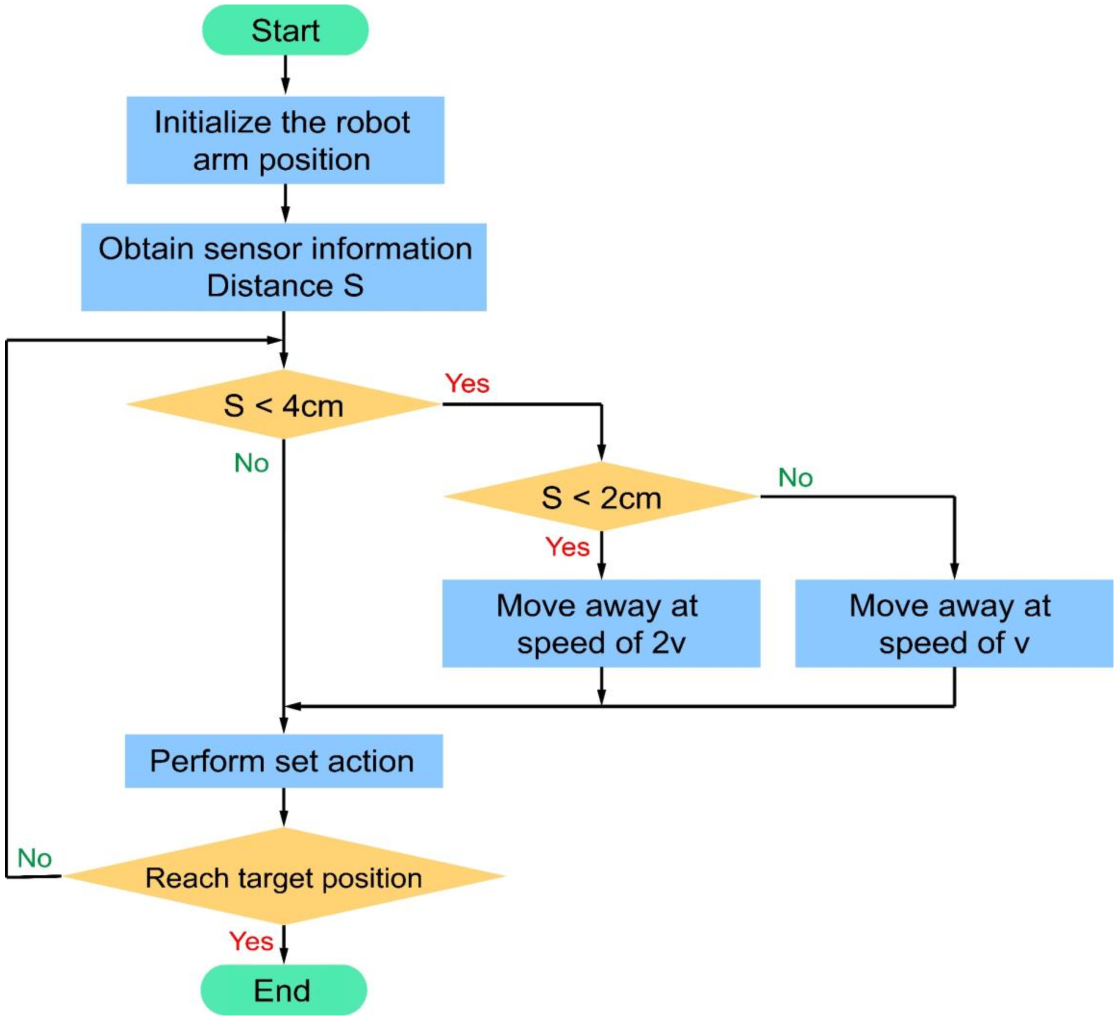

Figure S20. Schematic of the distance following control under different distances.

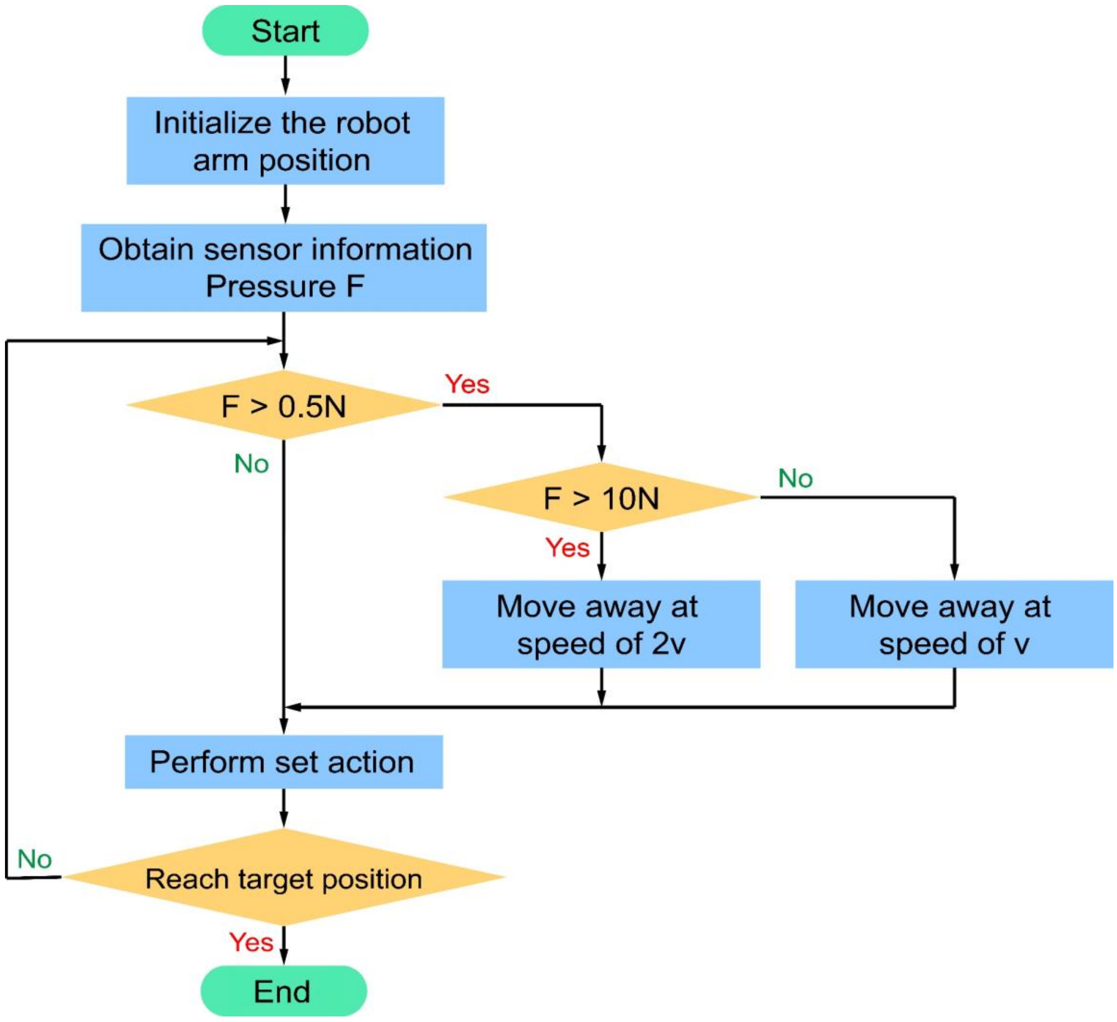

Figure S21. Schematic of the pressure following control under different pressures.

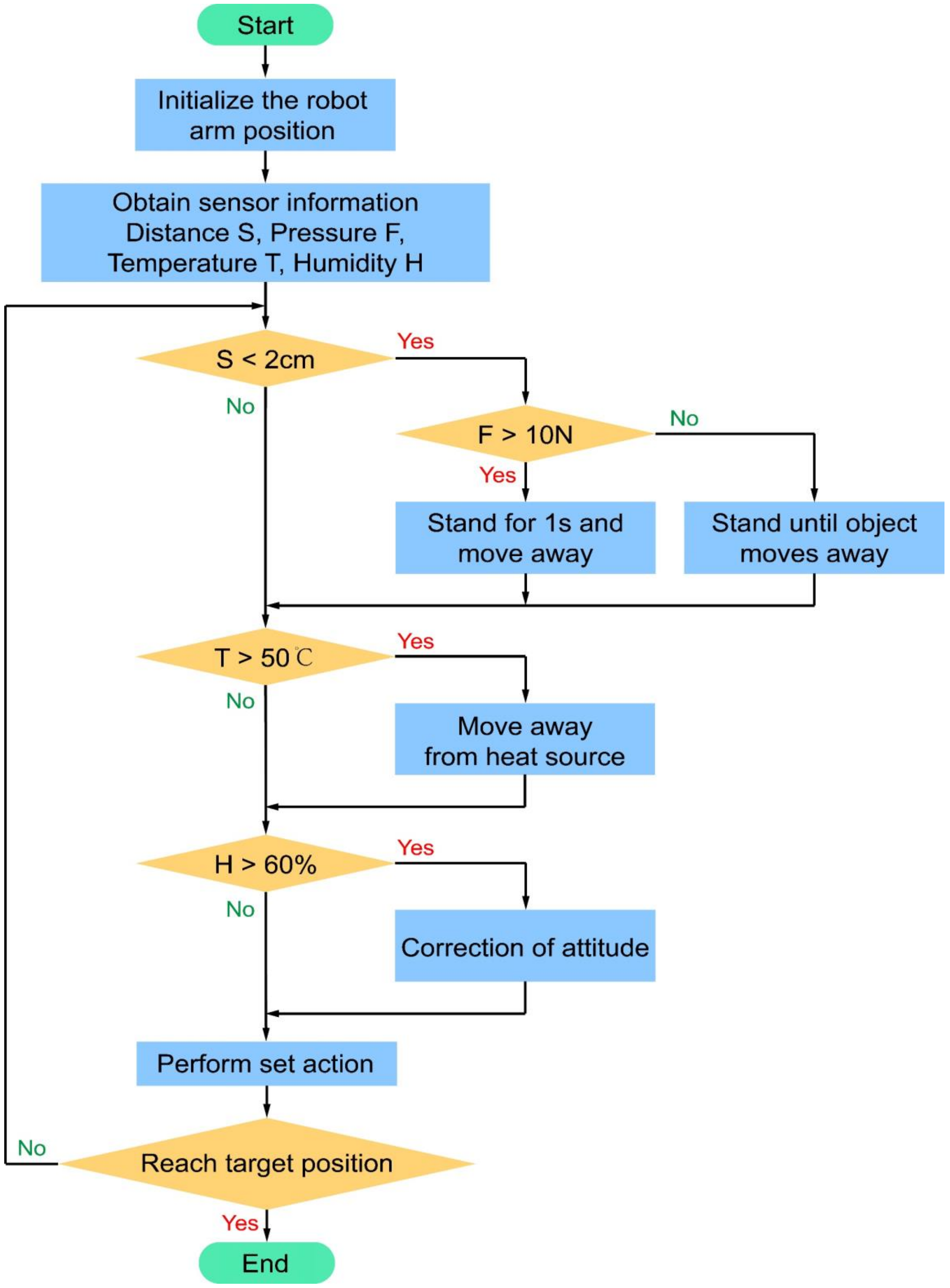

Figure S22. Schematic of the active safety control for various environmental changes.

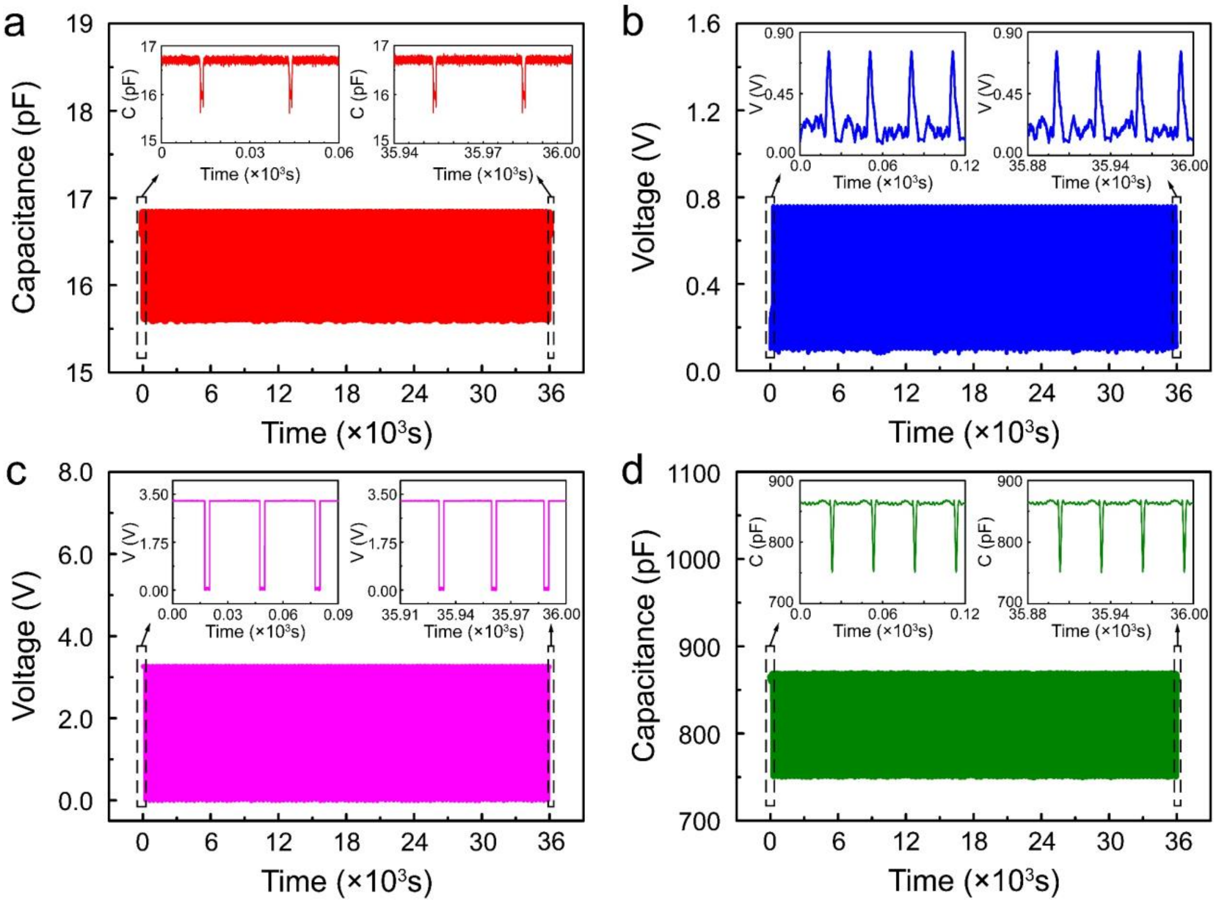

Figure S23. The output of sensor modules with distance, pressure, temperature and humidity fluctuating repeatedly on the robot arm, respectively.

Table S1: Comparison of sensors' performances.

Proximity measurement	Proximity sensitivity	Hysteresis error	Pressure measurement	Pressure sensitivity	Response time	Ref.
0~100mm	0.232mm^{-1}	0.75%	0~450 N	16.34 N^{-1}	70 ms	This work
5~50 mm	-	-	0~316kPa	-	-	[S1]
0~50 mm	-	-	0~360kPa	22.4 MPa^{-1}	-	[S2]
> 6 mm	-	-	0.1~10 N	-	5 ms	[S3]
0~200mm	-	-	0~200kPa	0.098kPa^{-1}	90 ms	[S4]
0~90 mm	-	-	0~300kPa	0.4 MPa^{-1}	-	[S5]

Table S2: Details of six sets of experiments.

Experiment	Type of object	Maximum approach velocity	Whether to use the LSTM network for classification or not	Repeat times
A	Conductors	18°/s	No	100
B	Conductors	18°/s	Yes	100
C	Semiconductors	18°/s	No	100
D	Semiconductors	18°/s	Yes	100
E	Insulators	18°/s	No	100
F	Insulators	18°/s	Yes	100

References:

[S1] H. S. Jo, S. An, C.-W. Park, D.-Y. Woo, A. L. Yarin, S. S. Yoon, *ACS Appl. Mater. Interfaces* **2019**, *11*, 40232.

[S2] Q. Hua, J. Sun, H. Liu, R. Bao, R. Yu, J. Zhai, C. Pan, Z. L. Wang, *Nat. Commun.* **2018**, *9*, 244.

[S3] J. Castellanos-Ramos, A. Trujillo-Leon, R. Navas-Gonzalez, F. Barbero-Recio, J. A. Sanchez-Duran, O. Oballe-Peinado, F. Vidal-Verdu, *IEEE T. Instrum. Meas.* **2020**, *69*, 4238.

[S4] Y. Qin, H. Xu, S. Li, D. Xu, W. Zheng, W. Wang, L. Gao, *IEEE Sens. J.* **2022**, *22*, 10446.

[S5] B. Zhang, Z. Xiang, S. Zhu, Q. Hu, Y. Cao, J. Zhong, Q. Zhong, B. Wang, Y. Fang, B. Hu, J. Zhou, Z. Wang, *Nano Res.* **2014**, *7*, 1488.
